# Nonallelic homologous recombination events responsible for copy number variation within an RNA silencing locus

**DOI:** 10.1002/pld3.162

**Published:** 2019-08-27

**Authors:** Young B. Cho, Sarah I. Jones, Lila O. Vodkin

**Affiliations:** ^1^ Department of Crop Sciences University of Illinois Urbana Illinois; ^2^ Present address: Carl R. Woese Institute for Genomic Biology University of Illinois Urbana Illinois

**Keywords:** copy number variation, *Glycine max*, nonallelic homologous recombination, RNA interference, soybean

## Abstract

The structure of chalcone synthase (*CHS*) gene repeats in different alleles of the *I* (inhibitor) locus in soybean spawns endogenous RNA interference (RNAi) that leads to phenotypic change in seed coat color of this major agronomic crop. Here, we examined *CHS* gene copy number by digital PCR and single nucleotide polymorphisms (SNPs) through whole genome resequencing of 15 cultivars that varied in alleles of the *I* locus (*I*, *i^i^*, *i^k^*, and *i*) that control the pattern distribution of pigments in the seed coats. Lines homozygous for the *i^i^* allele had the highest copy number followed by the *I* and *i^k^* cultivars which were more related to each other than to the lines with *i^i^* alleles. Some of the recessive *i* alleles were spontaneous mutations, and each revealed a loss of copy number by digital PCR relative to the parent varieties. Amplicon sequencing and whole genome resequencing determined that the breakpoints of several *i^i^* to *i* mutations resulted from nonallelic homologous recombination (NAHR) events between *CHS* genes located in segmental duplications leading to large 138‐kilobase deletions that erase the structure generating the *CHS* siRNAs along with eight other non‐*CHS* genes. Functional hybrid *CHS* genes (designated *CHS5:1*) were formed in the process and represent rare examples of NAHR in higher plants that have been captured by examining spontaneous mutational events in isogenic mutant lines.

## 1 INTRODUCTION

Repetitive sequences are extensive in many organisms including plant genomes. For example, 80% of the maize genome consists of repeats (Schnable et al., 2009) and regions of genome duplication have occurred in the progenitors of *Arabidopsis* (Arabidopsis Genome Initiative, 2000) and *Glycine max* (soybean), an inbred species of great agronomic importance for animal and human feed (Schmutz et al., 2010). Repetitive DNA regions in higher organisms can be the origin of chromosomal genomic rearrangements such as deletions, inversions, and insertions (Lye & Purugganan, 2019; Sasaki, Lange, & Keeney, 2010; Zmienko, Samelak, Kozlowski, & Figlerowicz, 2014). Repeated DNA regions often create problems in the assembly of short next‐generation sequence (NGS) reads that are typically 100–150 nucleotides (nt). This leads to mis‐assemblies and gaps in sequenced genomes (van Dijk, Jaszczyszyn, Naquin, & Thermes, 2018; Salzberg & Yorke, 2005; Treangen & Salzberg, 2012).

A repeated gene region in soybean is also an example of naturally occurring RNA interference (RNAi) that leads to a phenotypic change in the agronomically important trait of seed color. Chalcone synthase (CHS), the first committed enzyme in the pathway to synthesis of flavonoids and anthocyanins, has multiple family members in many plant species including soybean. Reduced *CHS* mRNAs and CHS activity were found in developing seed coats of soybean isolines with the dominant *I* (fully yellow seed coat and hilum, where the seed attaches to the pod) or *i^i^* (yellow seed coat with black hilum) alleles compared to varieties that are homozygous for a recessive *i* allele that specifies fully pigmented seed coats (Wang, Todd, & Vodkin, 1994). Sequenced recombinant lambda clones selected by hybridization with a CHS cDNA showed linkage of several *CHS* genes (*CHS1*, *CHS3*, and *CHS4*) within a 10.9‐kilobase (kb) segment (Akada & Dube, 1995) and conventional cloning and sequencing in several reports revealed at least nine *CHS* family members. By analyzing restriction fragment length polymorphisms (RFLPs) and polymerase chain reaction (PCR) products of spontaneous mutations of *I* → *i* or *i^i^* → *i* alleles, the *I* locus was identified to be a region of repeated *CHS* genes (Todd & Vodkin, 1996). Surprisingly, the presence of more *CHS* copies based on RFLPs using gene‐specific probes suppressed expression of *CHS* mRNAs while deletions of *CHS* genes restored higher *CHS* mRNA expression. In this regard, the dominant *I* alleles paralleled the phenomenon of cosuppression or post‐transcriptional gene silencing (PTGS) in plants (Napoli, Lemieux, & Jorgensen, 1990) that later was explained by the action of endogenous RNA interference (RNAi) by small RNAs (Baulcombe, 2004; Matzke & Birchler, 2005; Matzke & Matzke, 2004). In the soybean example, BAC (bacterial artificial chromosome) sequencing and NGS sequencing of small RNA populations showed that short interfering RNAs (siRNAs) related to *CHS* genes were produced in the Williams *i^i^* genotype (yellow seed coat with black hilum) by an unusual 27‐kb region that consists of two identical 10.91‐kb inverted repeats of *CHS1*, *CHS3*, *CHS4* and *CHS4*, *CHS3*, *CHS1* that are separated by 5.71 kb of intervening sequence that codes for one hypothetical protein (Clough et al., 2004; Tuteja, Clough, Chan, & Vodkin, 2004; Tuteja & Vodkin, 2008; Tuteja, Zabala, Varala, Hudson, & Vodkin, 2009). This region is referred to here as *CHS1‐3‐4‐Hypo‐CHS4‐3‐1* and is located on chromosome (chr) 8 along with six additional *CHS* genes. This unusual structure spawns tissue‐specific primary and secondary *CHS* siRNAs that regulate similar *CHS* mRNAs of which the unlinked and more distantly related *CHS7* and *CHS8* are the major downregulated targets in the immature yellow seed coats (Cho, Jones, & Vodkin, 2013; Tuteja et al., 2009).

Not only do the *CHS7* and *CHS8* genes produce the main transcripts involved in the seed coats that produce pigments in the homozygous *i* varieties, but they are also the main *CHS* genes involved in the production of the nutritionally significant isoflavonoids in the cotyledons that are encased within the seed coats (Dhaubhadel, Gijzen, Joy, & Farhangkhoee, 2007). Since the *CHS* siRNAs are tissue‐specific and produced only in the seed coats, the flavonoid pathway is not inhibited in the cotyledons and the isoflavonoids are still produced in soybean varieties with yellow seed coats. Similarly, the *CHS* genes, including those located on the chr 8 region, are more highly expressed or capable of induction in other tissues of *I* and *i^i^* genotypes, thus allowing production of other secondary products important for nodulation or plant defense in other tissues as roots and leaves. Mottling (irregular sectors of pigment) on otherwise yellow seed coats with *I* or *i^i^* genotypes has been shown to result from suppression of CHS PTGS by viral suppressor proteins (Senda et al., 2004) or from cold temperature stress (Nagamatsu et al., 2007).

In addition to the *I*, *i^i^*, and *i* alleles, there is a rare fourth allele, designated *i^k^*, that manifests a two‐colored seed coat. Known as the saddle pattern, the pigmented region occupies a saddle‐shaped region emanating from the hilum area to cover about two‐thirds of the seed coat. In addition to being produced in a tissue‐specific manner, the *CHS* siRNAs are also produced in a pattern‐specific manner within only the yellow sections of the seed coat proper (Cho, Jones, & Vodkin, 2017). The pigmented hilum and the saddle of seed with homozygous *i^i^* and *i^k^* genotypes have higher levels of *CHS7* and *CHS8* and low levels of *CHS* siRNAs, whereas the yellow sectors of the seed coat proper are the opposite with low *CHS* mRNA and high *CHS* siRNAs. We have also recently identified the unlinked *k1* mutation which interacts with the dominant *i^i^* to mimic the saddle pattern produced by the *i^k^* allele as a mutation in *Argonaute5*, a member of the small RNA pathway (Cho et al., 2017).

In this report, we have examined the *CHS* repeated region on chr 8 by digital PCR to assess copy number in varieties with different alleles of the *I* locus. We also subjected 15 of these lines (Data Set S1) to whole genome resequencing to define variation in the alleles of the *I* locus. We delineated the breakpoints of deletions in some rare recessive *i* mutations despite the fact that the first two public genome assemblies for soybean contained inversions or gaps and did not accurately represent the structure of this region because of the unusual repeated *CHS* gene arrangements within it. We found that at least three of the mutations result from nonallelic homologous recombination (NAHR) between some of the *CHS* gene repeats leading to large segmental deletions that erase the structure generating the *CHS* siRNAs along with eight other non‐*CHS* genes. Since the phenotype is easily observable, the black‐seeded *i* mutations represent rare examples of observed NAHR events in higher plants. Most examples of NAHR in higher plants have been inferred from gene arrangements as opposed to captured directly in mutational events (Zhang et al., 2015).

## 2 RESULTS

### 2.1 Current genome assemblies contain gaps in the *I* locus region which were corrected by BAC sequences

**Figure 1:**
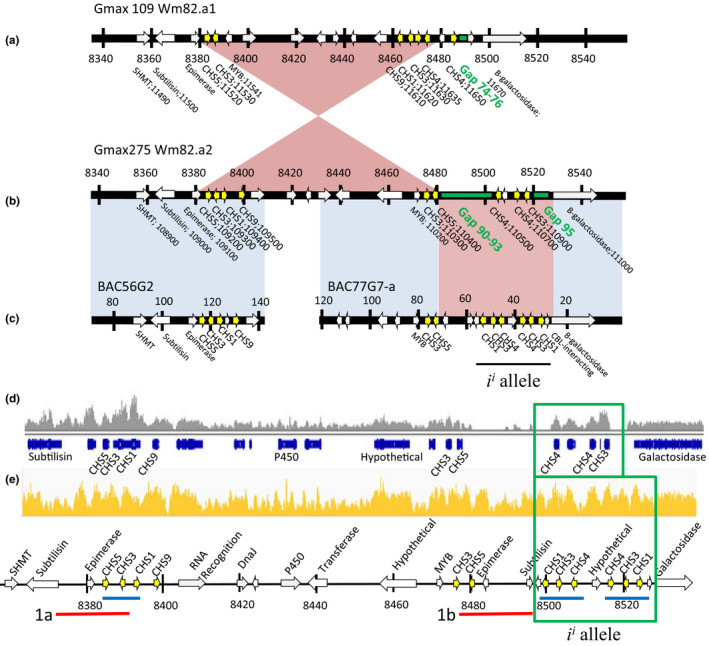
Genome organization around the *i^i^* allele containing highly repeated *CHS* genes. The current soybean reference genome (Gmax275 Wm82.a2) shows gaps in regions of highly repeated *CHS* genes when compared to the same region from BACs representing the *i^i^* allele. (a‐c) Genome segment of chr 8 near the *I* locus from a, the reference genome of the first soybean genome assembly Wm82.a1 (Schmutz et al., 2010) compared to (b), the current genome assembly in Wm82.a2 in Phytozome, and compared to (c), the indicated BAC (bacterial artificial chromosome) sequences from Clough et al. (2004) and Tuteja and Vodkin (2008) that were cloned from the same Williams 82 variety (*i^i^* allele) used in the two genome assemblies. Arrows indicate genes and direction. Yellow arrows are the 12 *CHS* genes present in this region. Green indicates gaps in the sequence that consist of runs of the base *N*. Select genes are labeled with only the numerical part of their Glyma gene identifier; for example, SHMT in part (a) is Glyma08g11490 and is Glyma.08G108900 in part (b). Triangles indicate an inverted region between two genome versions. Blue shading indicates the match between the reference genome and the regions of BAC56G2 and BAC77G7‐a, and the pink section indicates an incomplete area of the reference genome which is filled in by BAC sequences. Vertical bars on the gene tracks indicate 20‐kb segments with their positions in kb on the reference genome, and the region of the *i^i^* allele is indicated by a black line. (d) Genome browser view of the quantitative representation of each base (gray shaded area) resulting from alignments of Williams genomic resequencing data to the *i^i^* allele from the Gmax275 Wm82.a2 reference genome representing chr 8 from approximately 8,350,000 to 8,550,000 with the Glyma gene models from the browser outlined in blue beneath. The region of the *i^i^* allele is indicated by a green box. (e) Improved alignments (yellow shading) resulting from the same sequence data aligned against a modified genome fasta file in which the gapped regions of the Gmax275 Wm82.a2 sequence have been replaced with sequences from BAC77G7‐a. The annotations of that region are shown below and will be referred to subsequently as the modified genome representing the chr 8 region of the *i^i^* allele or *I* locus. The two red lines or three blue lines indicate areas of exact or very similar repeated sequences. The region of the *i^i^* allele is indicated by a green box

Alignments of whole genome resequencing data to the current soybean genome for Williams 82.a2 assembly available from Phytozome (https://phytozome.jgi.doe.gov/) contained multiple large gaps in the *I* locus region likely because of the repetitive nature of the *CHS* genes comprising the locus. We undertook a comparison of both assembly versions Gmax109 Wm82.a1 (released in 2010, Schmutz et al., 2010) and Gmax275 Wm82.a2 (released in 2012) for the soybean genome compared to several of our previously published BAC sequences (Clough et al., 2004; Tuteja & Vodkin, 2008) that were also derived from Williams 82. As shown in Figure 1, the two different genome assembly versions have a major inversion with respect to each other. Although the Wm82.a2 genome is more closely representative of our BAC sequences, there are still several large gaps in the *I* locus region where the genome was not assembled properly. Not surprisingly, the unusual 27‐kb inverted repeat structure of clusters of *CHS1‐3‐4* and *CHS4*‐*3‐1* genes previously found in our Williams 82 BAC sequences resides in those gapped regions found on chr 8. For this reason, we made a modification to the fasta file of the Williams 82.a2 chr 8 assembly, to fill in and correct that region. We then compared our genomic sequence data derived from the Williams cultivar with the Wm82.a2 genome and the modified genome (Figure 1d,e) and showed consistent high quality coverage with the *i^i^* allele region without any missing regions. In subsequent alignments, we primarily used the modified genome with the annotations of the *CHS* genes in that region as shown in Figure 1e with the addition of three *CHS* genes that form the 27‐kb inverted repeat *CHS1‐3‐4* and *CHS4‐3‐1* region and are the origin of the small RNAs from the *i^i^* allele. Correspondence of annotations of the other Glyma models within the 250‐kb region can be seen in Figure 1a,b including two other upstream clusters composed of *CHS5‐3‐1‐9* genes and another with *CHS3‐5*. All totaled, 12 CHS genes are found in this region with many of them in inverted orientation with respect to the adjacent *CHS* gene, although only nine are shown in the Wm82.a2 assembly. Very recently, a new soybean reference assembly version under construction, Wm82.a4, was made available at SoyBase (www.soybase.org). This version aims to fill in the gaps in the previous versions using PacBio BAC sequencing. This newer genome contains the full complement of 12 CHS genes on chromosome 8 in the arrangements as found in our previously sequenced BACs 56G2 and 77G7‐a as shown in Figure 1e and in our modified genome track of Figure 1e.

### 2.2 Digital PCR reveals copy number variation that distinguishes *I* locus alleles

**Figure 2:**
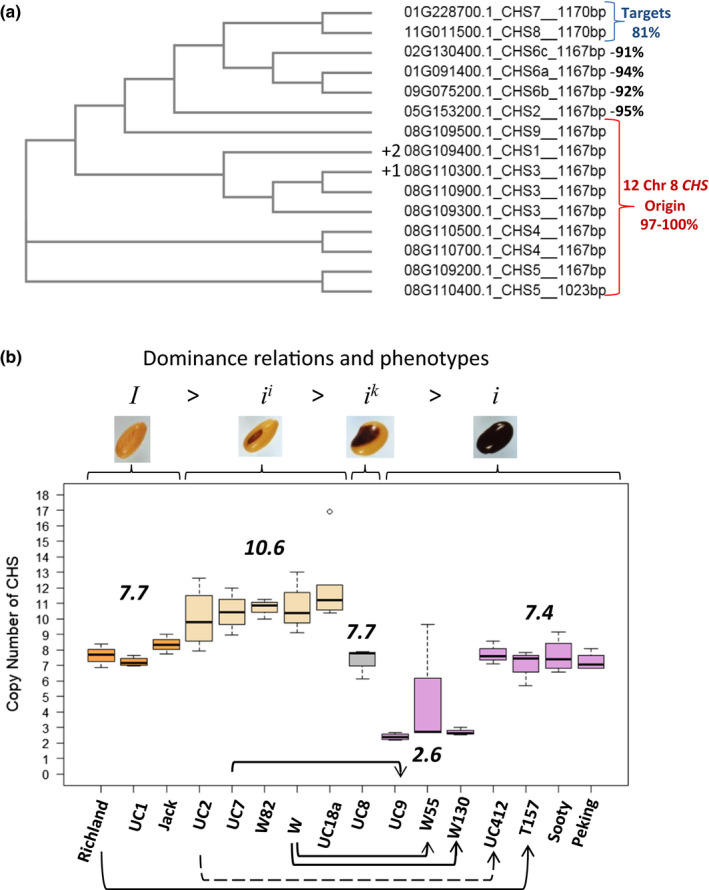
Digital PCR reveals copy number variation of *CHS* genes in lines with different alleles of the *I* locus. (a) Tree showing the relatedness of 18 *CHS* coding regions from the Williams 82 genome. The Glyma gene model number is shown as well as the specific *CHS* gene name and size of the mRNA coding sequence. The 12 repetitive *CHS* genes on chr 8 are marked in red, and the two unlinked target *CHS7* and *CHS8* genes are marked in blue. The +2 and +1 indicate the two extra identical copies of *CHS1* and one extra copy of *CHS3*, respectively, on the BAC and as shown in the modified genome of Figure 1 which do not have Glyma numbers. The 1,023‐bp *CHS5* was near the gap region and is slightly smaller in the Phytozome genome Glyma model but is intact in BAC77G7a. The percentage identities of the CHS coding regions are shown with respect to *CHS4*. The tree was constructed with the MUSCLE program at http://www.ebi.ac.uk/Tools/msa/muscle. (b) Standard box plots show copy number determinations of chr 8 *CHS* genes for three to six replicates of the indicated soybean cultivars on the x‐axis with different *I* alleles, grouped in order of their dominance relationships. The thick middle line of each box shows the median copy number. The average copy number for all lines within a genotype is indicated by the number in bold italics. UC lines are all isolines with various alleles that were introgressed into Clark as the recurrent parent. Solid line arrows represent spontaneous mutations derived from the indicated parent lines, and the dashed line arrow indicates an isoline relationship of UC2 (recurrent parent) to UC412 which carries two traits from an unadapted variety, an *i* allele specifying black seed coats and an unknown gene *def* specifying defective seed coats. The circle above Clark UC18a represents an outlier sample. See Figures S1 and S2 and Data Set S1 for pedigree relationships, full cultivar names, and other information. The identity matrix for the tree is shown in Data Set S2 and a Multalin of the sequences in Figure S3

We next assessed the organization of all other Glyma models within the Wm82.a2 assembly with PFAM annotations as “chalcone or stilbene synthase genes” in order to develop methods to assess copy number determination in various *I* locus alleles. A total of 24 Glyma models resulted, and a percent identity matrix with Clustal 2.1 is shown in Data Set S2. Three small gene fragments were annotated as Glyma models but we eliminated those from further analyses as well as six more distantly related genes (53% to 72% identity to *CHS4*) that may be members of the stilbene branch. The phylogenetic tree with the 18 remaining *CHS* gene models is shown in Figure 2. The 12 *CHS* genes on chr 8 (nine annotated Glyma models and the three *CHS* genes that fill in the gaps in the Wm82.a2 genome) all have 97%–100% identity with respect to *CHS4*. Except for the 12 on chr 8, none of the other six *CHS* genes are clustered. Additional *CHS* genes that have been annotated in the literature include a single copy of *CHS2* on chr 5 with 95% identity, and *CHS6* on chr 1 with 94% identity to *CHS4*. The genome contains two additional *CHS* genes annotated here as *CHS6b* on chr 9 and *CHS6c* on chr 2, which are 92% and 91% similar to *CHS4*. Finally, *CHS7* and *CHS8,* which are each 81% similar to *CHS4,* are found on chr 1 and chr 11, respectively. These two more distantly related genes are the targets of the small RNAs produced by the *CHS1‐3‐4* and *CHS4‐3‐1* inverted repeat region of the dominant *i^i^* allele.

In order to determine the copy number of the *CHS* genes in this region in lines with different alleles of the *I* locus, we used digital PCR with a Fluidigm BioMark™ instrument. CHS forward and reverse primers and reporter sequences are generic for the *CHS1, CHS3, CHS4, CHS5*, and *CHS9* genes located on chr 8, but have sufficient mismatches to distinguish those genes from *CHS2*, *CHS6*, *CHS7*, and *CHS8* which are located on other chromosomes (Table S1). The sequence of the lectin gene, *Le1*, (Vodkin et al., 1983) was used as a reference gene in soybean, as there is only one copy in the genome to which the chosen primers have similarity. The target *CHS* genes were labeled with FAM, and the single copy reference gene was labeled with VIC dye.

Figure 2b shows the results of copy number variation in 16 different soybean cultivars containing different alleles or mutations of the *I* locus. More description of the cultivars used is shown in Data Set S1. Several of them are isolines or mutations in a Williams background as shown in the pedigrees of Figure S1 or a Clark background as shown in Figure S2. The results of digital PCR demonstrated that there were clear groupings that corresponded to certain alleles of the *I* locus. For example, all five lines with *i^i^* genotype (yellow seed coat with black hilum) that produce the *CHS* siRNAs had the highest copy numbers, with an average median copy number for all five lines at 10.6 copies. This determination is similar to the known copy number of 12 *CHS* genes on chr 8 based on the modified Williams 82 genome as shown in Figure 1e. More interestingly, two derived spontaneous black seed coat mutations found in Williams isolines show dramatically reduced copy numbers of *CHS* genes: Line W55 (*i* black seed coat) had 2.7 copies and W130 (*i* black seed coat) had 2.6 copies. Similarly, UC7 (*i^i^* yellow with black hilum) with 10.4 copies spawned the mutation UC9 (*i* black) with 2.4 copies.

In addition to above‐mentioned mutations from *i^i^ → i* which clearly lost a number of *CHS* copies, there are some recessive *i* mutations that did not have as great a loss of copy number. For example, the isoline UC412 (*i* black) with 7.6 copies was formed by backcrossing for six generations with UC2 (*i^i^* yellow with black hilum) having 9.8 copies as the recurrent parent. Line T157 (*i* black) with 7.4 copies is a spontaneous mutation found in the cultivar Richland (*I* yellow with yellow hilum) having 7.7 copies indicating little, if any, change in *CHS* gene number as assayed with the primers used. The *I* allele has both a yellow seed coat and yellow hilum and also downregulates *CHS7* and *CHS8* genes through production of small RNAs by the *I* locus. We found similar copy numbers for three *I* alleles (Richland at 7.7 copies, UC1 at 7.1 copies, and Jack at 8.3 copies) which all apparently lack 2 or 3 *CHS* genes relative to the cultivars with *i^i^* alleles (yellow with black hilum). Interestingly, the *i^k^* allele (UC8, saddle pattern) and even *i* alleles from nonadapted small black‐seeded cultivars as Peking and Sooty had similar copy numbers as those of the three cultivars with the *I* (yellow with yellow hilum) alleles. All of the cultivars are homozygous for the *K1* allele that produces a functional *AGO5* gene except for UC18a which was discovered as a spontaneous mutation in the UC2 line and has *i^i^ k1* genotype, leading to a black saddle pattern which mimics the phenotype of the *i^k^ K1* allele in UC8 (Cho et al., 2017). As shown in Figure 2b, the UC18a line maintains the copy number of the parent UC2 line because they both have the *i^i^* genotype even though the seed phenotype is different.

### 2.3 Genomic resequencing reveals large deletions in several *i* (black) mutant alleles

**Figure 3:**
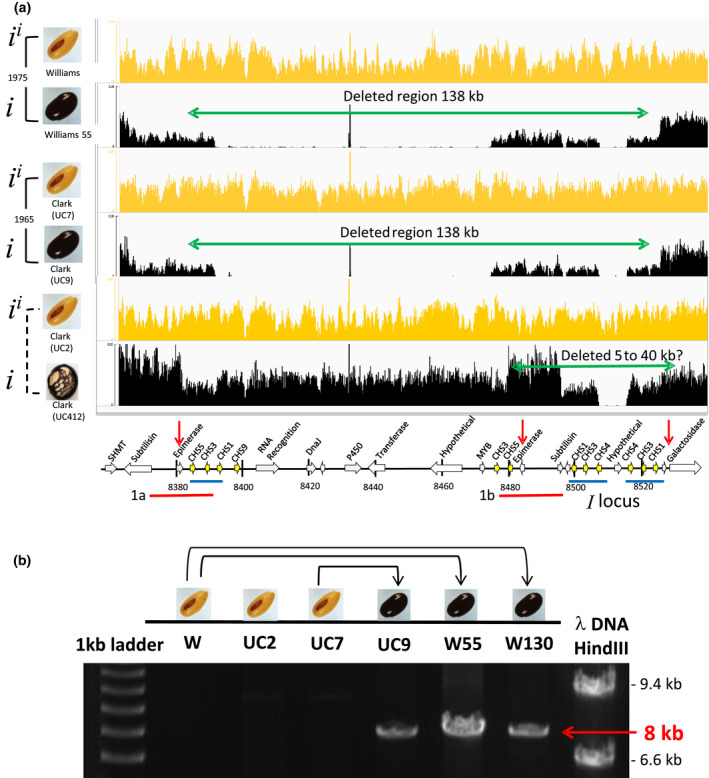
Genomic resequencing reveals large deletions in independent mutations of the *I* locus. (a) Representative images of seed phenotypes are shown for parent lines having the dominant *i^i^* allele with yellow seed coats or of the mutant lines with homozygous recessive *i* alleles that specify black seed coats. Direct isogenic mutants are indicated with solid lines and the year the mutation was discovered. The dashed line indicates an isoline relationship of the two varieties. Yellow or black graphs show normalized counts representing whole genome resequencing alignments against the modified genome in 0.25 scale [count at base × one million/total number of reads]. Red and blue lines represent identical or nearly identical regions. Red arrows indicate the location of primers used in PCR. Vertical bars on the gene tracks indicate 20‐kb segments with their positions in kb on the reference genome. Gaps in the mutant lines with the recessive *i* allele indicate missing areas of the genome where reads do not align except for those coming from repetitive regions including the *CHS* genes. Green arrows highlight the potential deleted region of 138 kb in the mutant lines Williams 55 and Clark UC9 as deduced from the PCR results shown in part (b). The deleted region of Clark UC412 is smaller, but its exact size is unknown due to the highly repetitive regions of inverted repeat *CHS* genes that flank the hypothetical gene in the center of the *i^i^* allele that aligns as if unique. Clark UC412 is an isoline with defective cracks in the seed coat that was created by repetitively backcrossing a Clark parent line with an unadapted variety PI 196166 that has both black seed coats and an unknown gene, *def*, that controls cracking of the seed coats. (b) An 8‐kb amplicon results from several independent mutations. PCR primers (Figure 3a) located 146 kb apart in the three yellow *i^i^* varieties were designed near the epimerase and galactosidase genes. The region is too large to be amplified in the yellow varieties but due to large deletions in several black mutant lines, a band of 8 kb results. The epimerase primer matches both inverted repeat regions 1a and 1b marked by red lines in Figure 3a; however, cluster 1b is apparently missing in the mutant lines, allowing the amplification from the epimerase primer annealing to cluster 1a with the unique galactosidase primer. Solid lines show the origin of the three black‐seeded mutant lines. W = Williams, and W55 and W130 are independent mutants derived from Williams. UC2, UC7, and UC9 are Clark isolines

Clearly, the copy number data confirm previous RFLP and PCR results showing that deletions around the *CHS* genes occurred in several of these spontaneous mutations. However, the digital PCR points to large‐scale loss of *CHS* genes in several of the *i^i^ → i* mutations. Alignments from paired‐end genomic resequencing data revealed large segments of missing sequence in the W55 mutation discovered in Williams in 1975 and in the independent UC9 mutation found in Clark in 1965 (Figure 3). The lack of alignments demonstrates that some of the non‐*CHS* genes within the *i^i^* allele region on chr 8 align as if unique in the soybean genome resulting in very few alignments that come from repetitive regions on other chromosomes. These include genes with annotations of RNA recognition motif, P450, transferase, myb factor, and two hypothetical genes. The *CHS1, CHS2, CHS3, CHS4, CHS5, CHS6,* and *CHS9* genes, on the other hand, are sufficiently similar to each other to show some alignments at each position with *CHS* gene reads that originate from either the chr 8 genes or other genomic locations. However, the level of base coverage is still substantially reduced within this region, likely from deletion of some of the genes. On the other hand, the isoline UC412 shows a much smaller deletion region which is only clearly observable in the genomic realignments because of the missing Glyma.08G110600 which encodes a hypothetical protein that is in the center of the loop of the 27‐kb *CHS1‐3‐4‐Hypo‐CHS4‐3‐1* inverted repeat which defines the *i^i^* allele.

Using a forward primer within the 5′ most epimerase gene (Glyma.08G109100) that is paired with a reverse primer in the 5′ region of the galactosidase gene (Glyma.08G111000) as shown in Figure 3a, we successfully amplified a fragment of approximately 8 kb in three lines: W55, UC9, and W130, the latter an independent mutation in Williams found in 1980 (Figure 3b). No band was found in the parent varieties because the fragment is too large to be amplified. The second primer binding position on the inverted repeated epimerase (Glyma.08G109100) does not interfere with the amplification because it is apparently deleted in the W55 and UC9 mutant lines as shown in Figure 3a. Based on the 8‐kb amplicon that resulted, we deduced that the deletion region is approximately 138 kb.

**Figure 4:**
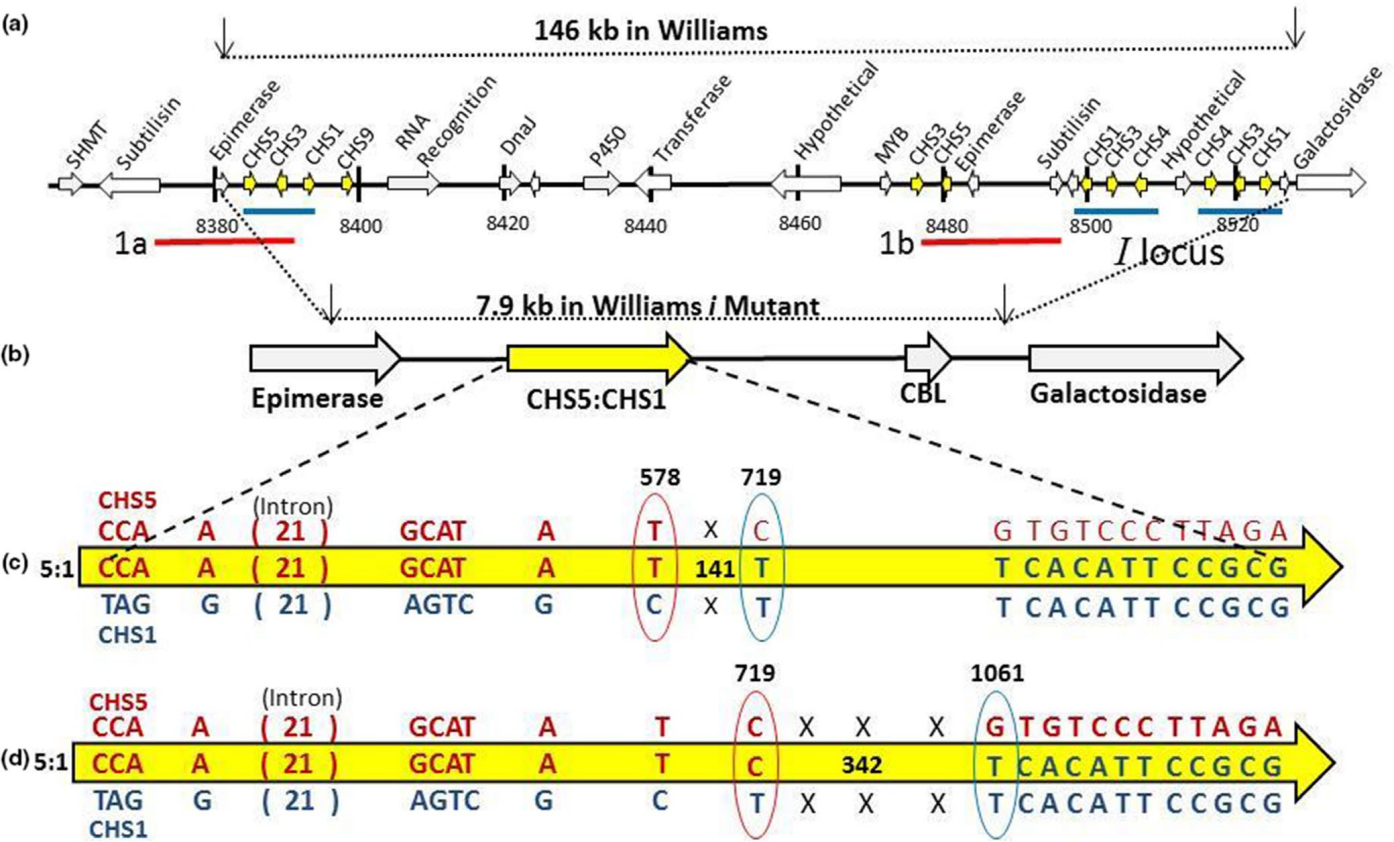
Deletions result in novel *CHS* genes by nonallelic homologous recombination events. A schematic of NGS sequencing of the 8‐kb amplicons shown in Figure 3b is presented. Full sequence data for alignments are shown in Figure S4 for the genomic, coding, and predicted protein sequences of the hybrid gene. (a) The modified chr 8 region near the dominant *i^i^* allele, as described in Figure 1. Black arrows represent positions of the primers which are 146 kb apart in the Williams *i^i^* genome. Red and blue lines represent repeated sequence regions. (b) Enlarged segment showing the structure of the 7,908 bp amplicons found in the three black seed coat lines with *i* deletion mutations. Black arrows correspond to arrows in part a representing the primer positions. The deletions result in a chimeric *CHS* gene, designated *CHS5:1*, an internal fusion of parts of two nonallelic but highly similar *CHS* genes. (c) Enlargement of the *CHS5:1* novel chimeric gene found in the W55 amplicon and showing the base differences in the gene. Numbers give the position from the ATG start, including the introns as shown in Figure S4a. Red indicates bases that match *CHS5*, and blue indicates bases that match *CHS1*. There are four mismatches within Exon 1 and 19 within Exon 2. The number of base differences within the intronic region is 21 total. A presumed recombination event denoted by X occurred somewhere within the 141 nt region between the circles marking positions 578 and 719 between which the switch from *CHS1* to *CHS5* occurred. (d) Enlargement of the hybrid *CHS5:1* chimeric gene found in both the UC9 and W130 mutant lines. In these lines, the recombination events occurred within a larger 342 nt region between positions 719 and 1061

To confirm the structure of the 8‐kb amplicon in the W55 mutation (*i*, black seed coats), it was subjected to Illumina sequencing and automated assembly protocols (without a reference sequence) at the MGH‐Harvard DNA Core facility. A total of 96,604 reads resulted allowing extremely high coverage of each base within the assembled 7,908 bp amplicon. Figure 4 shows a schematic of the alignment of this sequence to the modified genome in the region between the primers. As shown in Figure 4b, the amplicon sequence contained a single complete *CHS* gene which was found to be a hybrid between the first *CHS5* (Glyma.08G109200) and last *CHS1* (no Glyma number available) that is nearest the galactosidase gene. The *CHS1* has no Glyma number as it is one of the two missing *CHS1* genes in the Phytozome gene assembly in the region of the inverted repeat of the *i^i^* allele (Figure 1). This hybrid gene formed by the NAHR deletion event is referred to as *CHS5:1*, and Figure 4c summarizes the individual base differences found when it is aligned to the coding regions of *CHS5* and *CHS1* as shown in the full alignments using Multalin (Figure S4). The total number of differences between *CHS5* and *CHS1* was four in Exon 1, 21 within the intron, and 19 in Exon 2 including the stop codon. The red letters show the correspondence of the *CHS5:1* hybrid to *CHS5*, and the blue letters show the match of the *CHS5:1* gene to *CHS1*. Position 578 was the last base in which *CHS5:1* was definitely derived from *CHS5*, and position 719 was the first observable base where W55 switched from matching *CHS5* to matching *CHS1*. We can deduce that a recombination event between the two nonallelic *CHS5* and *CHS1* genes occurred somewhere in the 141 nucleotides between positions 578 and 719. Thus, amplicon sequencing confirmed a large deletion of 138 kb in the recessive *i* mutation to black seed coats and demonstrated that it arose from a nonallelic homologous recombination event between two similar but nonidentical *CHS* genes in tandem orientation with respect to each other.

Next, we sequenced the 8‐kb PCR amplicons derived from the two other spontaneous mutations, UC9 and W130, resulting in data sets of 228,668 and 213,458 reads, respectively. These two amplicons were identical to W55 except for a single base difference at position 719 with a transition of C to T (Figure S4). In this respect, these two amplicons were identical to *CHS5* at position 719 and did not resemble *CHS1* until the first difference at position 1061. Thus, the recombination events in these two *CHS5:1* hybrid genes were deduced to have occurred within the 342 bases between the positions 719 and 1061 in the genomic sequence.

Figure S4 shows Multalin outputs for all three mutations relative to *CHS5* and *CHS1* for the genomic sequences, coding sequences, and protein sequences. Although there were four differences in Exon 1 and 19 in Exon 2 of the transcript including one in the stop codon, there were only three amino acid differences between *CHS5* and *CHS1*. All three *CHS5:1* hybrid amplicons (W55, W130, and UC9) are similar to *CHS5* at amino acid positions 9 and 100 but the last amino at position 388 of the *CHS5:1* hybrid is now leucine like *CHS1* rather than valine as in *CHS5*. The single base difference of C to T between the three amplicons in the genomic sequence at position 719 and in the coding sequence at position 594 does not change the predicted amino acid sequences of the three *CHS5:1* hybrid genes with respect to each other as the C to T transition is in a synonymous codon for phenylalanine at position 198.

Numerous attempts with different primer sets were made to find an amplicon that spanned the smaller deletion in line UC412 (*i* black and defective seed coats) but none were successful. It is impossible to select primers that anneal uniquely within the identical repetitive regions near the *i^i^* allele (regions marked in red and blue in Figures 1, 3, and 4). Choosing primers that are clearly outside these regions (i.e., from the nonrepetitive myb gene anchored with the primer near the galactosidase which would produce a product of approximately 56 kb) also did not produce a product in long‐range PCR amplifications in either lines with the *i^i^* allele (yellow) or the UC412 (*i*) isoline. We speculate that the actual deletion is rather small in this line, knocking out the hypothetical gene as shown from alignments and possibly a couple of the *CHS* genes within the inverted repeats. The predicted size of the amplicon in this case is apparently still too large to be amplified in our reactions. The experimentally determined copy number of 7.6 for UC412 (Figure 2b) agrees with this interpretation.

### 2.4 The levels of SNPs define the *I* and *i^k^* alleles as distinct from the *i^i^* allele

**Figure 5:**
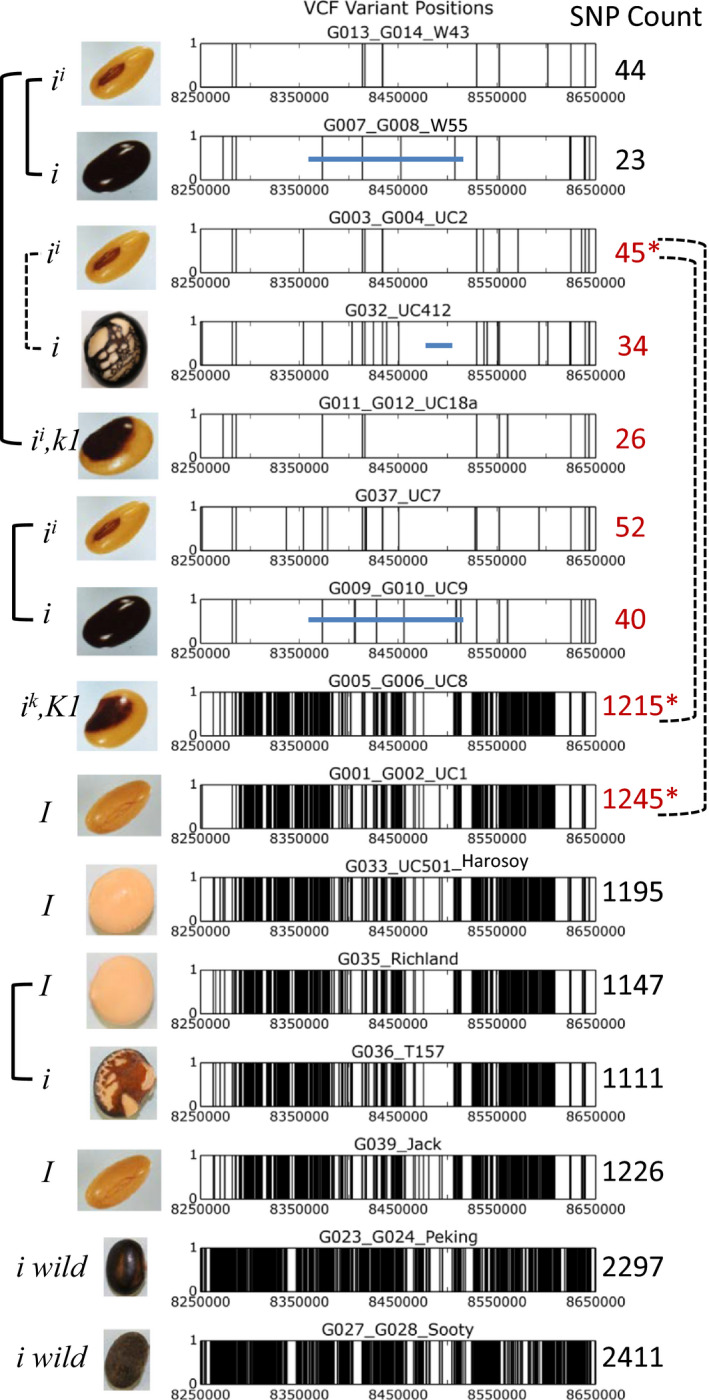
SNP variation within alleles of the *I* locus. Genotypes and seed phenotypes are shown on the left for each of 15 sequenced cultivars or isolines. Each vertical bar represents a single misalignment (SNP or small deletion/insertion) compared to the Williams 82.a2 reference genome (nonmodified) within a 400‐kb region of the *I* locus on chr 8 from positions 8250000 to 8650000. The blue horizontal line represents the approximate region of the 138‐kb deletion in the W55 and UC9 mutations and the smaller deletion region in UC412. Any SNPs within the deletion region represent alignments of a few sequences from similar regions on other chromosomes (see Figure 2). Solid lines on the right represent isogenic mutant pairs, and hatched lines represent backcrossed isolines with Clark (UC2) as the recurrent parent. The total number of SNP variants in the region is shown at the right and those in red indicate different alleles within the variety Clark. The large number of SNPs (linkage drag) observed in the Clark *I* and Clark *i^k^* isolines relative to the recurrent parent Clark (UC2) is marked with asterisks. Note: The defective seed coats of the T157 line results from an epistatic interaction of the *i* allele with the recessive *t* locus found in both Richland and T57 and encoding a flavonoid 3‐hydroxylase (Zabala & Vodkin, 2003) and not to the *def* gene for the net pattern defective seed trait that is present the UC412 line

We sequenced 15 cultivars having different alleles of the *I* locus, including seven isolines constructed with the same recurrent parent background (Clark) for six generations of backcrosses (Data Set S1). A table of variants at each position relative to the reference Williams 82.a2 genome was assembled for each variety. As shown in Figure 5 and Data Set S1, the variants were plotted to display SNP density (including small insertions and deletions) within a 400‐kb region from 8.25 to 8.65 million on chr 8 spanning the *I* locus. Williams is the variety most related to the Williams 82 (*i^i^*, yellow with black hilum) reference genome, and it showed only 42 variants within this 400‐kb region. The other three lines with the *i^i^* allele were in a Clark background and also have only 45, 26, and 52 variants. The W55 and UC9 spontaneous mutations of *i^i^* → *i* and the UC412 isoline with an *i* allele also had only a small number of variants. The variants within the deletion regions in these lines were from alignments of sequences from other repetitive regions in the genome.

In contrast to the *i^i^* alleles, three varieties homozygous for the dominant *I* allele (yellow with yellow hilum) had substantially larger numbers of variants with counts of 1,195 for Harosoy, 1,147 for Richland, and 1,226 for Jack. The spontaneous* i* mutation (black seed coat) that arose in Richland also had a large number of SNPs as expected. The large number of SNPs associated with the wider *I* allele region was carried as linkage drag when the *I* allele was introgressed into a recurrent parent Clark background as shown in the contrast of UC2 (Clark *i^i^*, yellow with black hilum) with 45 variants and UC1 (Clark *I* isoline, yellow with yellow hilum) with 1,245 variants. The SNP data also demonstrated that the *i^k^* allele (black saddle) is more similar to the *I* allele (yellow with yellow hilum) than to the *i^i^* allele (yellow with black hilum) as shown in the contrast of UC2 (Clark *i^i^*, yellow with black hilum) with 45 variants and UC8 isoline (Clark *i^k^,* black saddle) with 1,215 SNPs. A direct comparison of each of the 1,215 variants in UC8 (*i^k^* black saddle) and the 1,245 variants in UC1 (Clark *I*, yellow with yellow hilum) showed that 83% of them were a direct match. The two unadapted lines with *i* alleles, Peking and Sooty, each contained almost twice the number of SNPs within the region at 2,297 and 2,411, respectively. Figure S5 shows the numbers of SNPs for the entirety of chr 8 in bins of 10,000 nt. It is clear that the six generations of backcrossing to produce UC1 (Clark *I* isoline, yellow with yellow hilum) and UC8 (Clark *i^k^* allele, black saddle) introgressed a high SNP region of nearly 700 kb from chr 8 including the 8.25 to 8.65 M around the *I* locus as shown in Figure 5.

Display of alignment data from *I*, *i^k^*, and* i* wild alleles with the Integrated Genomics Viewer (IGV) browser showed complete base coverage without any evidence of large deletions at the *I* locus as for the W55, UC9, and UC412 mutant lines. However, there could be smaller deletions within the repetitive *CHS* genes as alignments will come from the other CHS gene regions. Direct evidence for a structural change of this nature deleting *CHS* genes is the copy number data of the *I*, *i^k^*, and *i* wild lines which all showed a lower copy number on the chr 8 region by three *CHS* genes as shown previously in Figure 2b. In order to determine that the SNPs in these lines did not interfere with the reporter and primer sequences used for digital PCR and lead to artificially lower copy numbers, we examined the variants within those regions within each *CHS* gene (Data Set S3). Of the 35 total variants across all 15 lines, only one SNP position was found in those regions and it was only present in two of the eight high SNP varieties that harbored either the *I*, *i^k^*, or *i* wild alleles (Figure 5). Finally, a recent reference quality assembly of a black‐seeded (*i* genotype) *G. soja* wild soybean line (Xie et al., 2019) contained eight *CHS* genes in the chr 8 locus region which is in good agreement with the digital PCR results for nonadapted Peking and Sooty varieties with *i* genotype (Figure 2b) and lower than the number of copies found in the dominant silencing *i^i^* allele with 11 *CHS* copies estimated by digital PCR (Figure 2b) and 12 copies found in the modified Wm82.a2 genome (Figure 1). The ancestral *G. soja* genome also is reported to contain an inversion relative to the domesticated Williams 82 cultivar sequences having the *i^i^* allele.

### 2.5 Genes within the deletion region were expressed in various tissues of the *i^i^* genotypes

**Figure 6:**
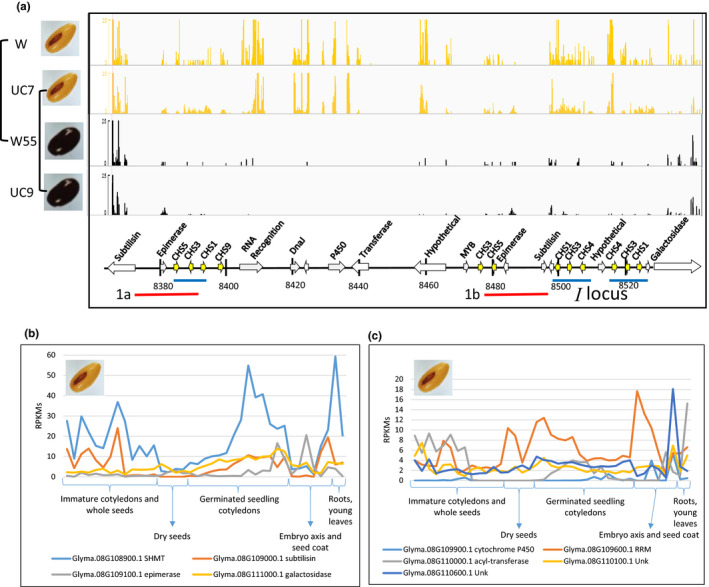
Comparative expression of transcripts within or flanking the 138‐kb deletion. (a) Alignments to the modified genome of RNA‐Seq data from immature seed coats of the 25–50 mg weight range from isogenic pairs, denoted by solid lines, for W, Williams (*i^i^*, yellow) and W55 (*i*, black mutation) or 100–200 mg from Clark UC7 (*i^i^*, yellow) and UC9 (*i*, black mutation). The same scale of 0.25 was used for display of all alignments [count at base x one million/ total number of reads]. The annotations of the *I* locus region are shown below as described previously in Figure 1. RNA levels expressed from exonic regions of the genes within the 138‐kb deletion region were substantially reduced, while the subtilisin and galactosidase flanking genes were apparently unaffected. (b‐c) Graphs of RNA‐Seq data isolated from 39 tissues and developmental stages from Williams (*i^i^*, yellow) with the major developmental or tissue groups marked. Transcript levels normalized in RPKMs for the four flanking genes are graphed in part (b) and those of five genes within the deletion region are shown in part (c). Unk, unknown function; RRM, RNA recognition motif. See Data Set S4 for the full data

Most of the eight non‐*CHS* Glyma models within the 138‐kb deletion region were expressed in the yellow seed coats of *i^i^* genotypes (Williams and UC7) but were not expressed in the black *i* mutant lines (W55 and UC9) as shown in the RNA‐Seq alignments of Figure 6 and RPKM data shown in Data Sets S4a,b. The expression of full‐length, non‐*CHS* genes immediately to the 5′ of the deletion event (subtilisin and epimerase) or to the 3′ of the deletion event (galactosidase) was not affected by the large deletion.

We next examined expression of the Glyma models flanking or within the *I* locus region in 39 different tissues and stages of immature seed and young seedling development of the Williams cultivar (*i^i^*, yellow with black hilum) as shown in Figure 6b,c and Data Sets S4a,c. Clearly, a number of the genes within the deletion region were expressed also in non‐seed coat tissues within the Williams (*i^i^*) as, for example, those representing a cytochrome 450, an acyltransferase, an RNA recognition motif, and two hypothetical genes of unknown function. Apparently, the absence of expression of these eight genes in the W55 (*i*, black) or UC9 (*i*, black) mutant lines that carries the 138‐kb deletion is tolerated without significant effects on overall plant growth and development. One of the most highly expressed models, Glyma.08G108900.1, approximately 25,000 bp to the 5′ side flanking the deletion breakpoint, encodes a serine hydroxymethyltransferase (SHMT), a gene recently identified as the *Rhg4* locus that confers resistance to the soybean cyst nematode (Liu et al., 2012). This gene was unaffected by the homologous recombination event between *CHS5* and *CHS1* that spawned the 138‐kb deletion (Data Set S4b).

## 3 DISCUSSION

### 3.1 Only the *CHS* genes on Chr 8 have extensive segmentally duplicated regions which differ in copy number variation in lines with different alleles of the *I* locus

Chalcone synthase is the first committed step in the pathway to a large array of diverse secondary products including compounds used to respond to biotic and abiotic stresses, exudates of the roots that are involved in nodulation and signaling, and pigments produced in the seed coats, pods, flowers, or trichomes. *CHS* genes have expanded to 18 members whose coding regions are clearly related ranging between 81% and 100% sequence identity (Figure 2a and Data Set S2) which allows for expression in various tissues or times of development. In addition, expansion of segmental duplication regions of the more closely related *CHS1, CHS3, CHS4, CHS5,* and *CHS9* genes that share >97% sequence identities on chr 8 created a new regulatory mechanism through endogenous CHS RNAi (Tuteja et al., 2004, 2009). This expansion leading to the 27‐kb *CHS1‐3‐4‐Hypo‐CHS4‐3‐1* inverted repeat is likely to be of relatively recent origin and associated with domestication, approximately 10,000 years ago. All commercially grown modern cultivars have one of the two dominant RNAi‐producing alleles of the *I* locus (*I* or *i^i^),* which inhibit production of the anthocyanin pigments on the seed coats despite the fact that most of the unadapted lines or wild relatives have pigmented seed coats. During soybean dehulling and processing of the protein isolates or oil for which soybean is valuable, the seed coat pigments can leave colored flecks or discoloration which is not desirable in the purified components. Thus, breeders incorporate the dominant alleles that produce predominantly yellow seed coats during cultivar development.

To assess copy number, we examined 16 varieties of soybean representing the four alleles of the *I* locus by digital PCR. The *i^i^* allele was found to have the highest copy number, while the *I* (yellow seed coat with yellow hilum) and *i^k^* (saddle seed pattern) alleles each have two to three fewer copies (Figure 2b). Whole genome resequencing of 15 of these 16 varieties also showed that the *I* and *i^k^* alleles are more similar to each other than to the *i^i^* allele. The *I* and *i^k^* alleles showed extensive SNP (including small indels) variation within 400 kb of the *I* locus that is carried as linkage drag when they replace the *i^i^* allele during six generations of recurrent backcrossing to an *i^i^* parent line with phenotypic selection for seed color (Figure 5 and Figure S5). As expected, the largest number of variants lies within the intergenic regions at 62.2% with 32.9% in the introns and 4.9% (200 total) within the coding sequences of the 51 Glyma models within that 400‐kb region (Data Set S3). Thus, when certain alleles of the *I* locus are introduced in breeding populations, a significant linkage drag can be expected when only the color phenotype is selected.

### 3.2 Dramatic copy number reductions from 138‐kb deletions formed by independent NAHR events generate hybrid *CHS* genes in at least three of the recessive *i* mutations

Back mutations from the *I* or *i^i^* alleles to full color (known as self‐color) occur infrequently, but have been found independently over 30 times in different cultivars, resulting in isogenic inbred lines that differ only at the *I* locus. It is now clear from the digital copy number determinations of several of these spontaneous mutations of *i^i^* → *i* genotype that they fall into different groups with variable numbers of copies deleted (Figure 2b). In three of them, the number of CHS genes was reduced from an estimate of 11 to only approximately two copies. Whole genome resequencing revealed that some of these *i^i^* → *i* isogenic lines exhibited very large deletions (Figure 3). NGS resequencing of the amplicons in three of the mutant lines proved that the origins of the deletions were independent nonallelic homologous recombination (NAHR) events between two *CHS* genes in the same orientation located 138 kb apart on chr 8 (Figure 4). The NAHR events reduced the copy number from 12 *CHS* genes on chr 8 to just a single functional gene that was clearly composed of a hybrid between the highly similar *CHS5* and *CHS1* genes that originally were 138 kb apart (Figure 4). The effect of this large deletion is that eight other soybean genes are deleted, apparently without major impact on the growth and development of the plant.

A specific 2.4‐kb Hind III RFLP fragment representing the promoter region of *CHS4* (out of at least eight visible bands in the complex coding region CHS RFLP patterns) was previously found to be absent in genomic DNA blots of the black seed coat mutant lines W55 and UC9 as well as five other independent mutant lines out of ten total that were examined (Todd & Vodkin, 1996). The 2.4‐kb fragment can only be absent when both of the identical *CHS4* genes in the *CHS1‐3‐4‐Hypo‐CHS4‐3‐1* inverted repeat of the *i^i^* allele are deleted. From resequencing of the UC412 isoline, it is apparent that a much smaller region was deleted and it included the hypothetical gene found in the center of the inverted repeat region of the *i^i^* allele (Figure 3a). A smaller deletion region, just in the vicinity of the 27‐kb inverted repeat clusters, agrees with the digital PCR (Figure 2b) indicating two to three *CHS* genes may be missing in UC412 (*i*, black seed coats). Thus, different NAHR events potentially occurred within the segmentally duplicated *CHS* regions that lead to variable size deletions in some of the independent *i* alleles or mutations.

Four of the *I* → *i* isogenic line pairs, including Richland (*I*, yellow) and T157, a spontaneous black‐seeded mutation found in Richland in 1936, were also previously examined for RFLPs. In contrast to the *i^i^* RFLP patterns, each of the *I* alleles contained a new Hind III fragment of approximately 12.1 kb which also hybridized with a specific probe for the *CHS1* promoter region (Todd & Vodkin, 1996). The 12.1‐kb fragment was missing in the T157, and other *i* mutant lines which implied structural changes, likely deletions, were taking place near a *CHS1* gene. Several reports using inverse PCR amplifications from genomic DNA of Japanese variety Miyagi shirome with *I* (yellow) genotype or its spontaneous mutation to *i* (pigmented) have indicated structural changes around a duplicated *CHS1* gene and a closely linked truncated *CHS3* partial pseudogene in inverted orientation (Kasai, Kasai, Yumoto, & Senda, 2007; Senda et al., 2002). Here, digital PCR of Richland and two other cultivars with *I* alleles demonstrated that their *CHS* gene copy numbers were lower by two to three copies relative to the 12 *CHS* genes of the *i^i^* allele (Figure 2b) While resequencing data revealed considerable SNP variation (Figure 5), alignments to the reference genome did not show any obvious lack of coverage implying that these eight non‐CHS gene sequences including the hypothetical gene (Glyma.08G110600) were present in the *I* genotypes.

### 3.3 Few NAHR mutational events have been described in higher plants

While the mechanistic process of NAHR has been delineated in model systems such as yeast, its effect on genome instability in humans and other higher organisms has been well documented (Sasaki et al., 2010). Transposable elements and LINES (long interspersed repetitive elements) can occur in high or low copy numbers. Another class of repeats known as LCR (low copy repeats or segmental duplications) comprises 5% of the human genome and is defined as repetitive regions of DNA >1 kb that share >90% identity. There are numerous examples of human genome neurological or developmental disorders with severe consequences that originated from gene duplications or deletions that can range from a few kb to nearly 2 Mb in size and that have resulted from NAHR events between LCR regions (reviewed in Sasaki et al., 2010).

Copy number variation (CNV) in plants has recently gained importance along with SNPs as a generator of intraspecies genome variation. While there are assessments of overall levels of CNV from comparative genome hybridizations or whole genome sequencing studies of different cultivars or ecotypes in several species including *Arabidopsis*, rice, maize, sorghum, wheat, potato, and soybean, only a few cases show clear phenotypic differences that relate to the copy number variation (reviewed in Zmienko et al., 2014 and Lye & Purugganan, 2019). One of the defined examples of phenotypic differences is for the ESPS gene, for which there were 10 to 40 more copies within some *Aramanthus* species that are more resistant to the herbicide glyphosate (Gaines et al., 2010). Another example is the *Rhg1* locus in soybean that controls resistance to the soybean cyst nematode with increasing copy number of a genomic segment containing three different genes (Cook et al., 2012) and another is a reproductive morphology locus in cucumber that results from a duplication segment containing four genes (Zhang et al., 2015). A recent review cites 25 examples of CNV having a role in domestication or diversification genes in plants (Lye & Purugganan, 2019) including *Tunicate1* (pod corn) in maize (Han, D., & Martienssen, R., 2012; Wingen et al., 2012) and the *Sh1* (seed shattering) genes of rice and sorghum (Lin et al., 2012). The *I* locus in soybean represents an LCR region that displays *CHS* gene copy number variation between genotypes. In contrast to the phenotypic change being the consequence of increasing copy number of the *CHS* structural genes and their enzymatic products, it is the unusual arrangement of some of the extra copies that is critical to induce RNAi and thus lead to the yellow seed coat in the domesticated cultivars. Thus, this arrangement was likely created by gene expansion during early domestication of soybean and preservation by early agricultural societies as well as modern breeders. Whether or not the *I* locus is classified as a true domestication trait, it is clearly a diversification gene, as are alleles coding for coat color in sheep or cattle (Lye & Purugganan, 2019).

Generation of deletions (Weil & Wessler, 1993; Yu, Zhang, Pulletikurti, Weber, & Peterson, 2010) and duplications (Zhang, Zuo, & Peterson, 2013) has been shown to occur at relatively high frequencies by aberrant movement of transposable elements, as detected by their effects on maize seed pigmentation traits that are easy to score. Rates for NAHR events in humans occur at lower frequencies of 10^–5^ to 10^–6^ (Sasaki et al., 2010), but only a few reports have determined rates experimentally in higher plants. In *Arabidopsis*, recombination between two nonfunctional 1.2‐kb partial copies of an overlapping sequence of a luciferase reporter gene that were separated by 4 kb was observed to reconstitute a functional reporter gene at rates of 10^–4^ to 10^–6^ (Molinier, Ries, Bonhoeffer, & Hohn, 2004). Careful assessment of structural variation in whole genome sequencing studies of an individual can infer that variants arise by nonallelic homologous recombination by examining the sequences adjacent to the presumed breakpoints for regions of homology. Using these criteria, only 0.7% of the 11,891 deletions in the cucumber genome were inferred to arise from nonallelic homologous recombination mechanisms which is much less than the 23% calculated for the human genome (Zhang et al., 2015). In cucumber, most of the deletions arose from nonhomologous rearrangement events and from transposable‐element‐mediated events.

While some of the occurrences of CNV in plants are assumed to arise by recombination between repeated regions, few have been shown to arise from NAHR by direct examination of de novo mutations. Because of the effect on color phenotype, the back mutations from yellow to black represent easily observable NAHR events in the large fields or breeding populations of soybeans, although the exact rates for these events are not known. By examining the structure of recessive *i* mutations, we captured several independent products of rare NAHR events in which hybrid *CHS* genes were formed between two *CHS* genes located 138 kb apart with the loss of 11 *CHS* genes within the deletion region. The loss of the eight non‐*CHS* genes is apparently well tolerated since their absence does not lead to other observable phenotypic differences in the *i* plants with black seed coats. Future unambiguous assemblies within particular repetitive regions will allow examination of more of the CNV in different cultivars or individual progeny or mutations and will likely include more integration of BAC sequence data as in this report, or by expanded use of longer reads from single molecule sequencing methods (reviewed in Goodwin, McPherson, & McCombie, 2016) as used for the repetitive maize zein genes (Dong et al., 2016) and for improving the maize genome assembly (Jiao et al., 2017) and a recent assembly of a wild soybean *G. soja* genome (Xie et al., 2019).

In summary, we have previously shown that an expanded region of *CHS* genes on chr 8 that forms the *CHS1‐3‐4‐Hypo‐CHS4‐3‐1* inverted repeat also spawned production of *CHS* siRNAs resulting in yellow seed coats that were selected during domestication (Cho et al., 2013; Tuteja et al., 2009). In this report, we showed that the lack of *CHS* siRNAs in several naturally occurring pigmented mutations results from independent NAHR events which delete many of the *CHS* genes within that unusual repeat structure to form hybrid *CHS* genes. Copy number determinations and SNP densities also distinguish other alleles of *I* locus into different groups.

## 4 METHODS

### 4.1 Plant materials and DNA and RNA extraction

The soybean (*Glycine max*) lines used in this study are inbred and homozygous for the indicated loci. All lines were developed by soybean geneticists and breeders during the 1960s or after and are available from the USDA germplasm collection through GRIN (Germplasm Resources Information Network). The full list of lines used in this report is shown in Data Set S1 and pedigrees in Figures S1 and S2. DNA was extracted from young shoot tips as described previously as were RNAs from the indicated tissues or stages of development (Cho et al., 2017).

### 4.2 Digital PCR and analysis of *CHS* copy number variation

Customized TaqMan primers and one reporter were prepared from Applied Biosystems. We prepared two sets of primers: one for the *CHS* genes (target) and the other for *Lectin1* (reference) which has only one copy in the soybean genome (Vodkin, Rhodes, & Goldberg, 1983). The target gene is labeled by FAM, and the reference gene is labeled by VIC dye. DNA samples were fragmented to 3 kb by M220 Focused‐Ultrasonicator™ (Covaris). Digital PCR was performed with Fluidigm BioMark™ (Fluidigm) by the Keck Center (University of Illinois) following the Fluidigm protocol. Results were analyzed by Fluidigm Digital PCR Analysis software. The software performed a statistical analysis to find the 95% confidence intervals of the true concentrations and the ratio of two concentrations (Dube, Qin, & Ramakrishnan, 2008). Three to six biological replicates were used for each variety.

### 4.3 Whole genome resequencing and data analyses

Whole genome sequencing library construction and high‐throughput sequencing were performed by the Keck Center (University of Illinois). The libraries were prepared with Kapa Library Construction Kits (https://www.kapabiosystems.com) and quantitated by qPCR and sequenced on one lane for 100 cycles from each end of the fragments on a HiSeq 2000 (Illumina) using a TruSeq SBS sequencing kit version 3 and analyzed with Casava 1.8 (pipeline 1.9). The average size of the DNA fragments was around 500 nt; insert size is 300 nt. The total reads ranged from 317 to 750 million per sample yielding ample coverage for the 1‐Gb genome. Quality checks for sequencing data were done by FastQC (Andrews, 2010). Alignments were performed by using Bowtie 2 (Langmead & Salzberg, 2012). We used default alignment and reporting options. Bowtie 2 output files, in SAM format, were converted to the BAM format and sorted by Samtools (Li et al., 2009). The Integrative Genomic Viewer (IGV) was used to convert from BAM to TDF format and visualize normalized (count at base × one million/ total number of reads) data (Robinson et al., 2011). To call SNPs, we used the Samtools command (mpileup‐uf) and converted BCF to VCF format to visualize in IGV using either the Gmax275 Wm82.a2 soybean reference (https://phytozome.jgi.doe.gov) or the file modified to correct the sequence around the *i^i^* allele as described in Figure 1. A database of all unique single nucleotide polymorphisms (SNPs) and small insertions and deletions was assembled from all of the VCF files from the Bowtie 2 alignments against the Wm82.a2 genome. This allows comparison of these variants across any cultivar or set of cultivars.

### 4.4 Amplicon sequencing and analyses

Amplicons that span the deletion were amplified from genomic DNAs extracted from shoot tips of W55, W130, and UC9 mutant lines (*i*, black seed coats). The primers used are forward primer BC1F (5′CTATAATCATATCTATGGACTCTCCCTC3′) paired with a reverse primer BC2R (5′AACTTTAACCTCTCATGTAAGGAAACAAATAAC3′). BC1F is located in the 5′ most epimerase gene (Glyma.08G109100) and BC2R is located in the 5′ region of the galactosidase gene (Glyma.08G111000).

Polymerase chain reactions were performed in 50 μl volume reactions in 0.2 ml Corning Axygen thin‐walled tubes with 8 μl of 2.5 mM dNTP mix, 1 μl each BC1F and BC2R primers at 10 mM, and 0.5 μl of 5 units/μl of Takara LA Taq HS (contains Mg2+). Amplifications were performed in a PTC‐200 DNA Engine from MJ Research using reaction times as follows: initial denaturation step at 94°C for 1 min followed by 29 cycles of denaturing at 94°C for 10 s, annealing at 55°C for 1 min, and elongation at 68°C for 9 min, to end with a 10‐min extension at 72°C. For each soybean genotype, four reactions were pooled and purified with a Zymo DNA Clean and Concentrator kit and the concentration adjusted to between 40 and 64 ng/μl. A total of 35 μl were submitted for barcoding of PCRs, amplicon sequencing with the Illumina MySeq, and automated assembly by the Center for Computational and Integrative Biology DNA Core Facility at Massachusetts General Hospital. Coverage of each amplicon was 96,604 reads for W55; 213,458 for W130; and 228,668 for UC9. Representation of each base in an assembled contig sequence was generally >1,000 with the great majority being perfect matches to the consensus contig. Blast alignments were made to BAC56G2, BAC77G7‐a, or a 300,000 bp segment from the modified genome. Multiple alignments were conducted with Multalin (Corpet, 1988).

### 4.5 Messenger RNA sequencing and data analyses

Analysis of transcriptome data for *I* locus regions was derived from our previously published data sets on immature cotyledons or seed coats (Jones & Vodkin, 2013) and seedling cotyledons (Shamimuzzam & Vodkin, 2014) from the Williams cultivar. Unreported libraries representing nine additional tissues or developmental stages of immature seed or seed coats, or roots and leaves from seedlings were added to make a set of 39 different libraries representing multiple stages and repeats of tissues from the Williams cultivar. Also, four libraries comparing *i^i^* versus *i* alleles are reported here. Methods of library construction and sequencing were previously described and are summarized in Data Set S4. The total number of reads ranged from 19.5 to 183 million. All libraries were aligned using Bowtie 1 (Langmead, Trapnell, Pop, & Salzberg, 2009) to the transcripts representing all 88,647 Glyma models including all splice variants allowing three mismatches and up to 25 alignments per read. Hits were normalized as RPKM (reads per kb of model per million mapped reads) as in Mortazavi, Williams, McCue, Schaeffer, & Wold, 2008. Glyma models representing the first splice variant and genes within or flanking the *i^i^* allele were selected from the data and plotted in Excel graphs representing their expression trends during development. Alternatively, alignments of mRNA sequences against the modified Wm82.a2 were performed by using Bowtie 1. Alignments were made with no mismatches allowed by the command (‐v 0). We used commands (‐best ‐‐strata ‐M 1) that chose one among multiple valid alignments, which had a 100% match, and randomly distributed it (Treangen & Salzberg, 2012). Output files in SAM format were converted to the BAM format and sorted by the Samtools program (Li et al., 2009). The Integrative Genomic Viewer (IGV) was used to convert from BAM to TDF format and visualize normalized (count at base × one million/ total number of reads) data (Robinson et al., 2011).

### 4.6 Accession numbers

The data supporting the findings in this paper are shown in Data Sets [Link], [Link], [Link], [Link] and include the Short Read Archive accession numbers for all whole genome sequencing analyzed including 13 cultivars reported here, GenBank accessions for the novel *CHS5:1* hybrid amplicons from W55 (MK603183), W130 (MK658865), and UC9 (MK673308), and GEO (Gene Expression Omnibus) series GSE123655 for 12 new RNA‐Seq samples reported here.

## CONFLICT OF INTEREST

The authors declare no conflict of interest associated with the work described in this manuscript.

## AUTHOR CONTRIBUTIONS

Y.B.C. and S.I.J. collected tissue samples, performed extractions, designed and conducted experiments, analyzed and interpreted data, and drafted results; L.O.V. designed approaches, obtained funding, led and coordinated the project, interpreted and analyzed data, drafted sections, and edited the manuscript.
