# Supplementary material for: Nonallelic homologous recombination events responsible for copy number variation within an RNA silencing locus
Source: Plant Direct. 2019 Aug 27;3(8):e00162. doi: 10.1002/pld3.162 (PMC6710647; doi:10.1002/pld3.162)
Supplement: Supplementary file 1 [file PLD3-3-e00162-s005.pdf]

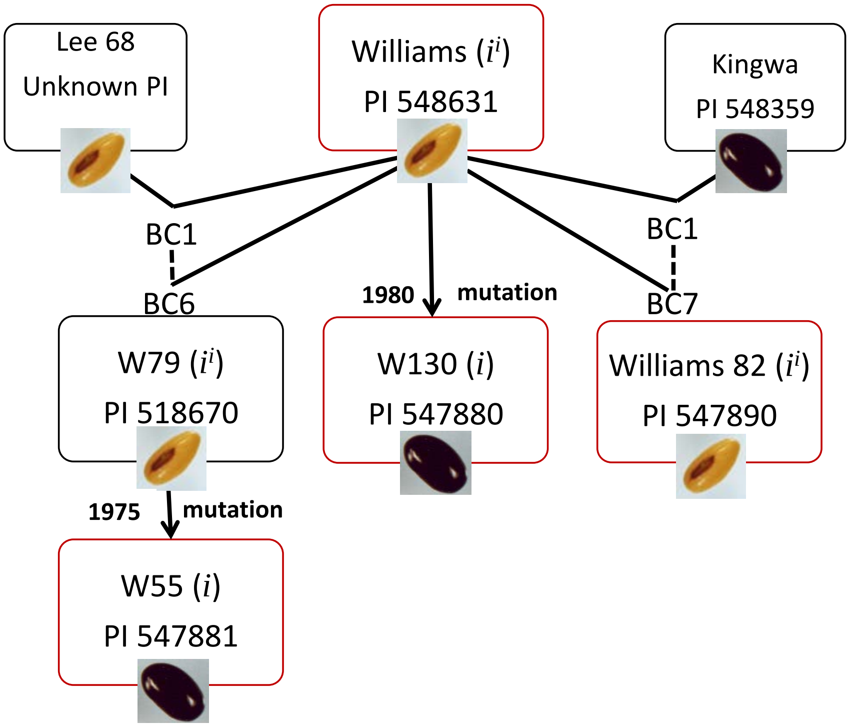

Supplemental Figure 1. Pedigrees of Williams Isolines Used in This Report.

Seed icons show the color phenotypes specified by alleles of the *I* locus. Pedigree information is available from the USDA Germplasm Resources Information Network (GRIN) which is searchable by the Plant Introduction (PI) number. Additional information and lab numbers for the lines used in this report (red outline) are listed in Supplementary File 1. Arrows indicate spontaneous mutations, while solid and dotted lines indicate backcrossing (BC). For example, W55 is a black seeded mutation that occurred spontaneously in the Williams 79 cultivar in 1975 and W130 was found in Williams in 1980. Williams 79 and Williams 82 are isolines created by bringing in disease resistance genes with 6 or 7 rounds of backcrossing (BC) using Williams as the recurrent parent.

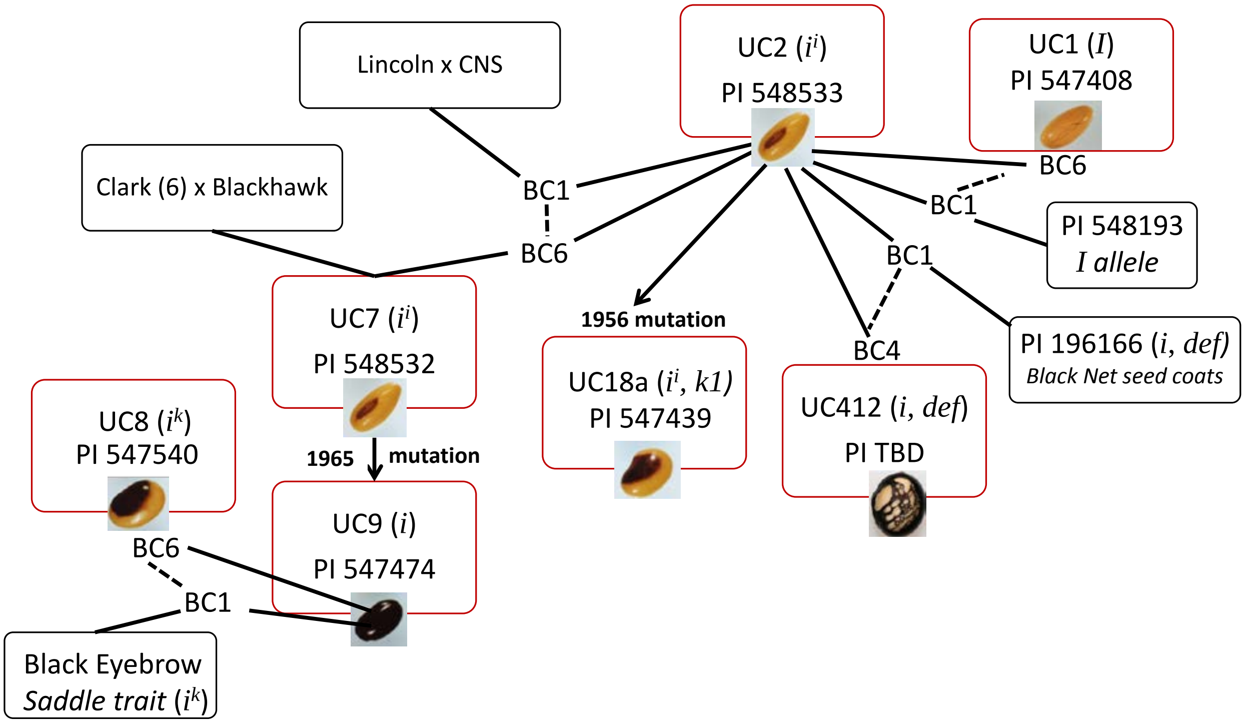

Supplemental Figure 2. Pedigrees of Clark Isolines Used in This Report.

Pedigrees of Clark isolines used in this report. Seed icons show the color phenotypes specified by alleles of the *I* locus. Pedigree information is available from the USDA Germplasm Resources Information Network (GRIN) which is searchable by the Plant Introduction (PI) number. Additional information and lab numbers for the lines used in this report (red outline) are listed in Supplementary File 1. Arrows indicate spontaneous mutations while solid and dotted lines indicate backcrossing (BC).

Clark 63 (UC7) was created by bringing in disease resistance genes with 6 rounds of backcrossing (BC) using Clark (UC2) as the recurrent parent. UC9 is a spontaneous black seed coat (*i*) mutation that was found in Clark 63. UC9 was used as the recurrent parent to create line UC8 (with the two color saddle phenotype) that carries the i^k allele of the *I* locus in place of the *i* allele. UC18a arose as a spontaneous mutation of the *K1* to *k1* allele which also creates a saddle pattern similar to the i^k allele (UC8). UC1 was created by backcrossing the dominant *I* allele into Clark to produce completely yellow seed coats. UC412 was created by backcrossing the recessive *i* and *def* genes into Clark to produce black defective seed coats (also called a net pattern) with defects in the cell walls of the seed coats.

Supplemental Data Cho et al.

Supplemental Figure 3. Multiple alignment (Multalin) of coding sequences of the 15 CHS genes. The Glyma number from the Phytozome Wm82.a2 genome is shown on the right of the indicated *CHS* gene.

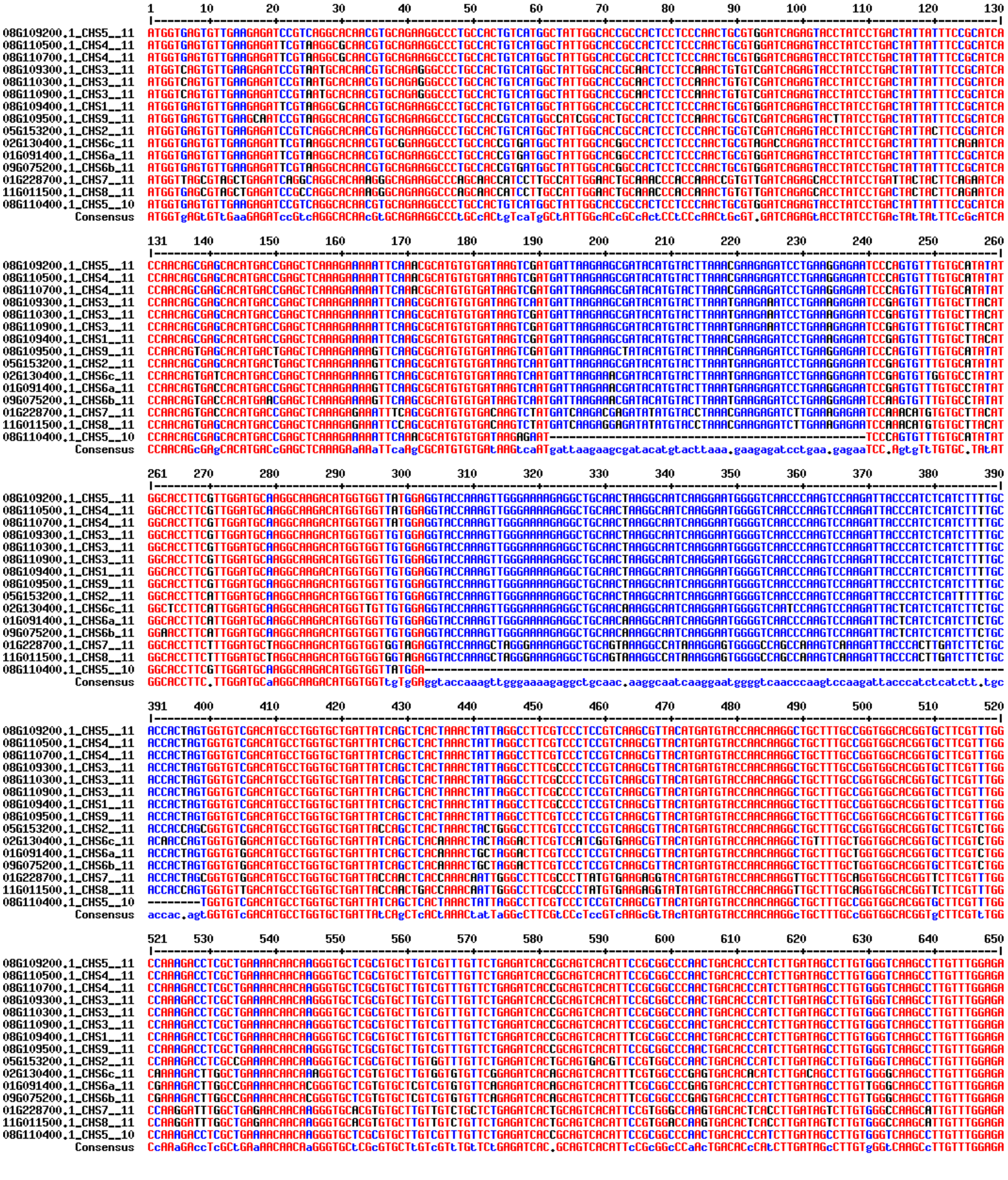

Supplementary Fig. 3 continued on next page

Supplementary Fig.3, continued.
Multiple alignment (Multalin) of coding sequences of the 15 CHS genes

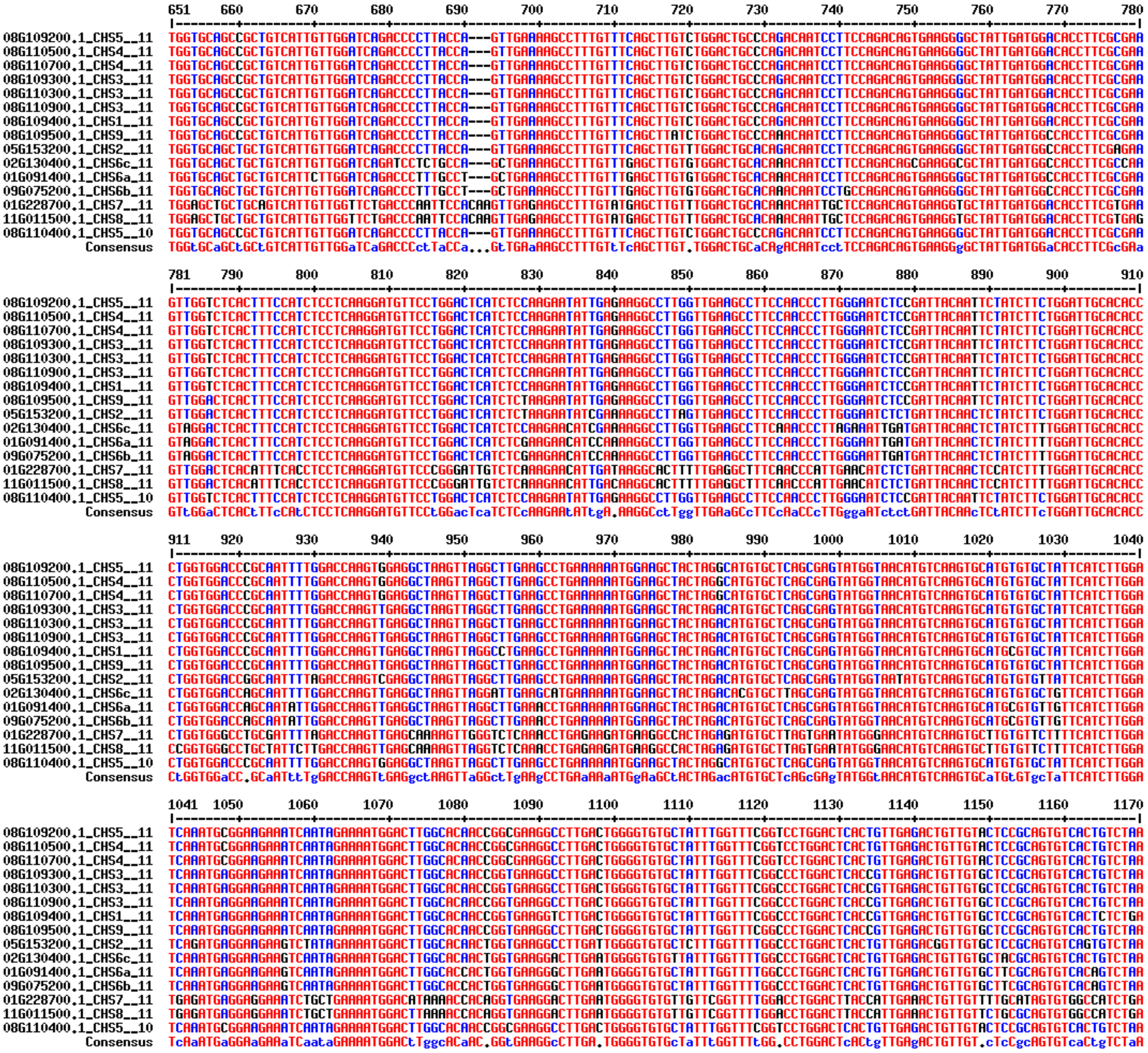

Supplementary Fig. 4a. Alignment of genomic sequences for the three *CHS5:1* hybrid amplicons relative to *CHS5* and *CHS1* using Multalin. The single intron is marked by the green line above the numbers. In W55 for example, the number of differences between *CHS5* and *CHS1* are 4 in Exon 1, 21 within the intron, and 19 in Exon 2. The square box indicates the single base difference within the three *CHS5:1* hybrids in the W55, W130, and UC9 mutant lines at position 719 which is also the first observable base where W55 switches from matching *CHS5* to matching *CHS1*. The dotted box marks position 1061 where W130 and UC9 switch from matching *CHS5* to matching *CHS1*.

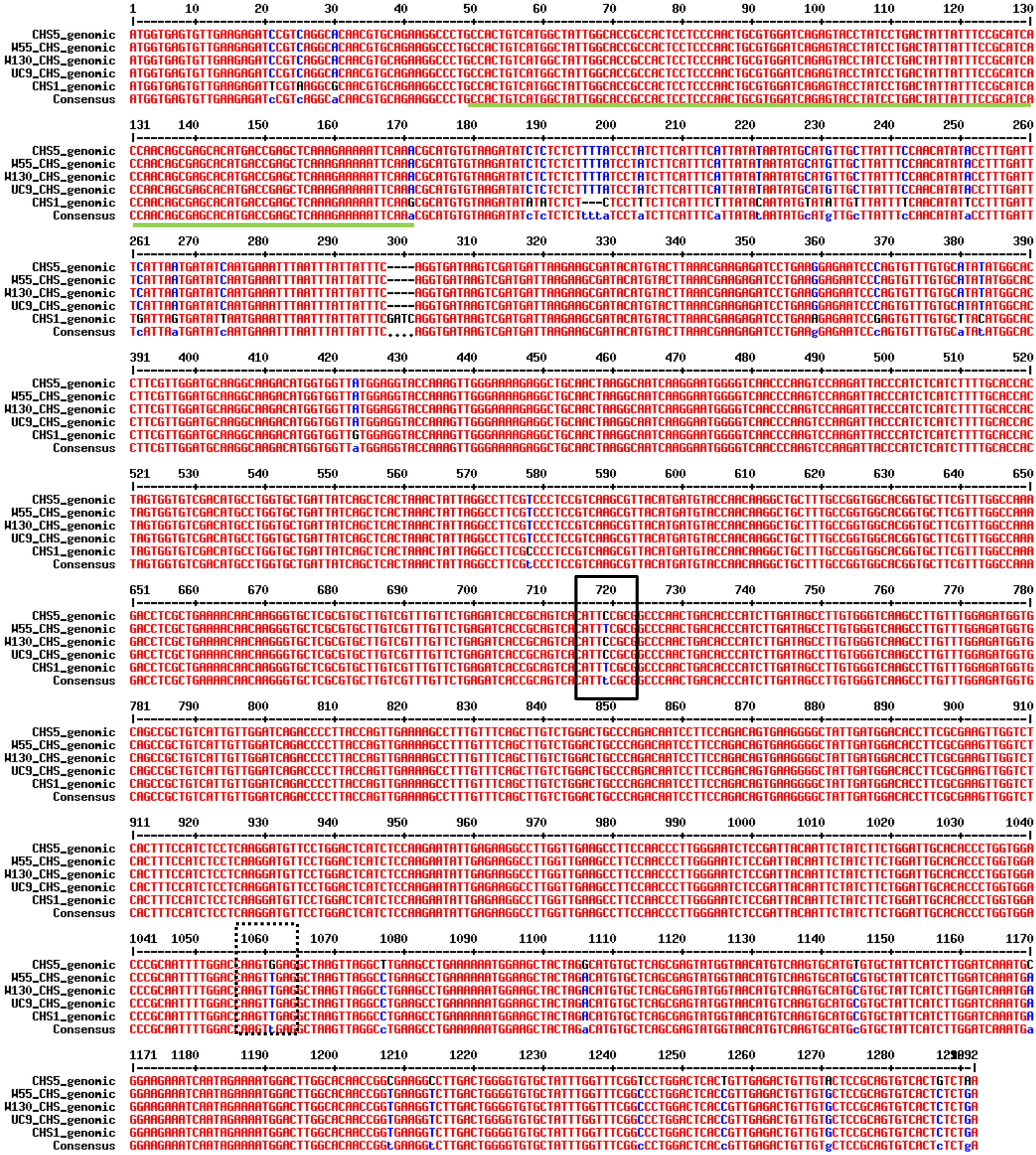

Supplementary Fig. 4b. Alignment of coding sequences for the three *CHS5:1* hybrid amplicons relative to *CHS5* and *CHS1*. There are 4 nucleotide differences between *CHS5* and *CHS1* in Exon 1 and 19 in Exon 2 including in the stop codon. The box marks the single base difference between W55 compared to W130 and UC9 at position 594 in the coding sequence.

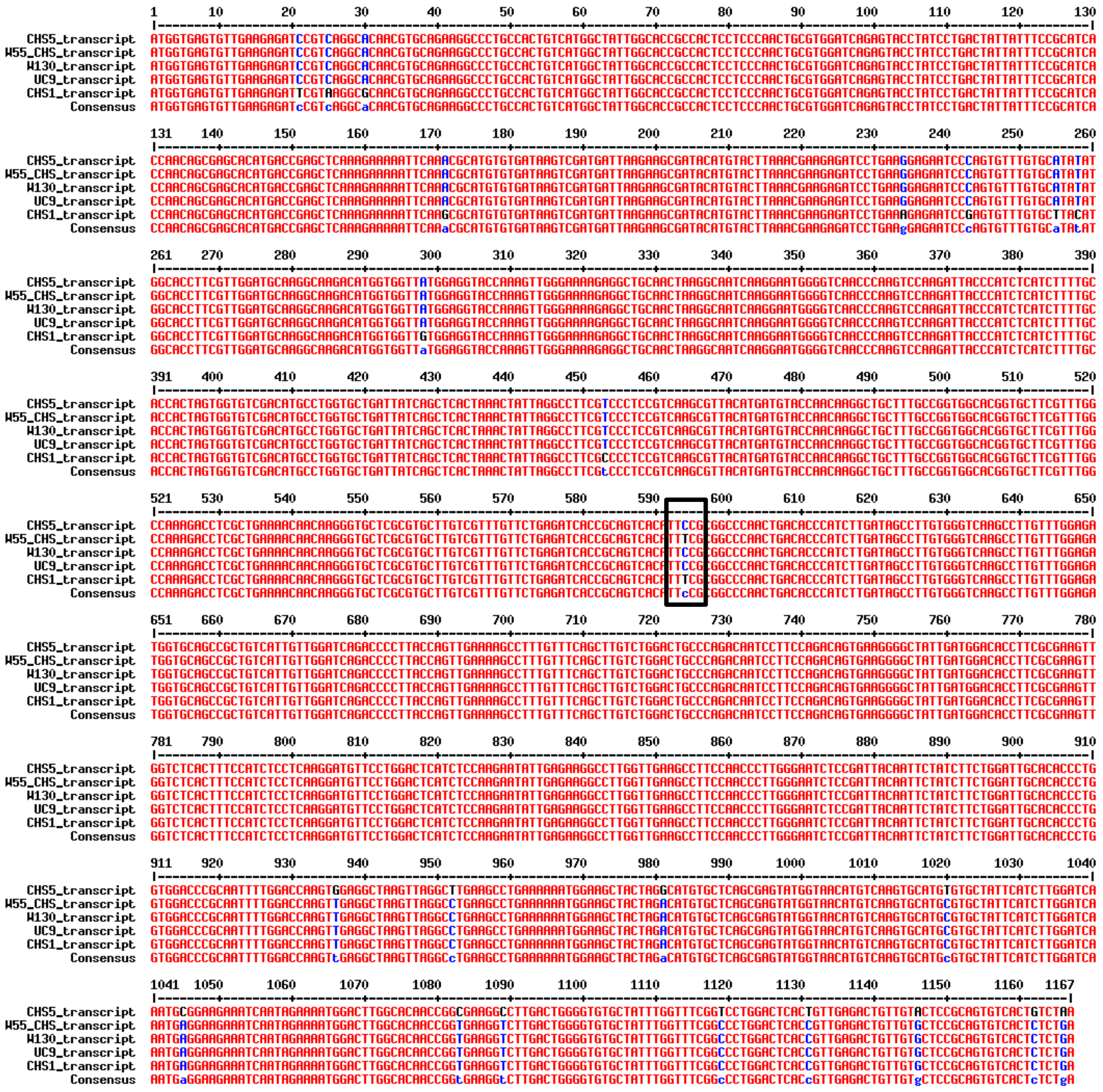

Supplementary Fig. 4c. Alignment of amino acid sequences for the three CHS5:1 hybrid amplicons relative to CHS5 and CHS1 proteins. Only three amino acids differ between CHS5 and CHS1. All three CHS5:1 hybrid amplicons (W55, W130 and UC9) are similar to CHS5 at positions 9 and 100 but the last amino acid switches to be similar to CHS1 at position 388. The single base difference of C to T between the three amplicons in the genomic sequence at position 719 and in the coding sequence at position 594 does not change the predicted amino acid composition of the CHS5:1 hybrid gene as it is a synonymous codon for phenylalanine at position 198.

```
                 1       10        20        30        40        50        60        70        80        90        100       110       120       130
   CHS5_protein  MVSVEEIRQAQRAEGPATVMAIGTATPPNCVDQSTYPDYYFRITNSEHMTELKEKFKRMCDKSMIKKRYMYLNEEILKENPSVCAYMAPSLDARQDMVVMEVPKLGKEAATKAIKEWGQPKSKITHLIFC
W55_CHS_protein  MVSVEEIRQAQRAEGPATVMAIGTATPPNCVDQSTYPDYYFRITNSEHMTELKEKFKRMCDKSMIKKRYMYLNEEILKENPSVCAYMAPSLDARQDMVVMEVPKLGKEAATKAIKEWGQPKSKITHLIFC
   W130_protein  MVSVEEIRQAQRAEGPATVMAIGTATPPNCVDQSTYPDYYFRITNSEHMTELKEKFKRMCDKSMIKKRYMYLNEEILKENPSVCAYMAPSLDARQDMVVMEVPKLGKEAATKAIKEWGQPKSKITHLIFC
    UC9_protein  MVSVEEIRQAQRAEGPATVMAIGTATPPNCVDQSTYPDYYFRITNSEHMTELKEKFKRMCDKSMIKKRYMYLNEEILKENPSVCAYMAPSLDARQDMVVMEVPKLGKEAATKAIKEWGQPKSKITHLIFC
   CHS1_protein  MVSVEEIRKAQRAEGPATVMAIGTATPPNCVDQSTYPDYYFRITNSEHMTELKEKFKRMCDKSMIKKRYMYLNEEILKENPSVCAYMAPSLDARQDMVVVEVPKLGKEAATKAIKEWGQPKSKITHLIFC
      Consensus  MVSVEEIRqAQRAEGPATVMAIGTATPPNCVDQSTYPDYYFRITNSEHMTELKEKFKRMCDKSMIKKRYMYLNEEILKENPSVCAYMAPSLDARQDMVVmEVPKLGKEAATKAIKEWGQPKSKITHLIFC

                 131     140       150       160       170       180       190       200       210       220       230       240       250       260
   CHS5_protein  TTSGVDMPGADYQLTKLLGLRPSVKRYMMYQQGCFAGGTVLRLAKDLAENNKGARVLVVCSEITAVTFRGPTDTHLDSLVGQALFGDGAAAVIVGSDPLPVEKPLFQLVWTAQTILPDSEGAIDGHLREV
W55_CHS_protein  TTSGVDMPGADYQLTKLLGLRPSVKRYMMYQQGCFAGGTVLRLAKDLAENNKGARVLVVCSEITAVTFRGPTDTHLDSLVGQALFGDGAAAVIVGSDPLPVEKPLFQLVWTAQTILPDSEGAIDGHLREV
   W130_protein  TTSGVDMPGADYQLTKLLGLRPSVKRYMMYQQGCFAGGTVLRLAKDLAENNKGARVLVVCSEITAVTFRGPTDTHLDSLVGQALFGDGAAAVIVGSDPLPVEKPLFQLVWTAQTILPDSEGAIDGHLREV
    UC9_protein  TTSGVDMPGADYQLTKLLGLRPSVKRYMMYQQGCFAGGTVLRLAKDLAENNKGARVLVVCSEITAVTFRGPTDTHLDSLVGQALFGDGAAAVIVGSDPLPVEKPLFQLVWTAQTILPDSEGAIDGHLREV
   CHS1_protein  TTSGVDMPGADYQLTKLLGLRPSVKRYMMYQQGCFAGGTVLRLAKDLAENNKGARVLVVCSEITAVTFRGPTDTHLDSLVGQALFGDGAAAVIVGSDPLPVEKPLFQLVWTAQTILPDSEGAIDGHLREV
      Consensus  TTSGVDMPGADYQLTKLLGLRPSVKRYMMYQQGCFAGGTVLRLAKDLAENNKGARVLVVCSEITAVTFRGPTDTHLDSLVGQALFGDGAAAVIVGSDPLPVEKPLFQLVWTAQTILPDSEGAIDGHLREV

                 261     270       280       290       300       310       320       330       340       350       360       370       380     388
   CHS5_protein  GLTFHLLKDVPGLISKNIEKALVEAFQPLGISDYNSIFWIAHPGGPAILDQVEAKLGLKPEKMEATRHVLSEYGNMSSACVLFILDQMRKKSIENGLGTTGEGLDWGVLFGFGPGLTVETVVLRSVTV
W55_CHS_protein  GLTFHLLKDVPGLISKNIEKALVEAFQPLGISDYNSIFWIAHPGGPAILDQVEAKLGLKPEKMEATRHVLSEYGNMSSACVLFILDQMRKKSIENGLGTTGEGLDWGVLFGFGPGLTVETVVLRSVTL
   W130_protein  GLTFHLLKDVPGLISKNIEKALVEAFQPLGISDYNSIFWIAHPGGPAILDQVEAKLGLKPEKMEATRHVLSEYGNMSSACVLFILDQMRKKSIENGLGTTGEGLDWGVLFGFGPGLTVETVVLRSVTL
    UC9_protein  GLTFHLLKDVPGLISKNIEKALVEAFQPLGISDYNSIFWIAHPGGPAILDQVEAKLGLKPEKMEATRHVLSEYGNMSSACVLFILDQMRKKSIENGLGTTGEGLDWGVLFGFGPGLTVETVVLRSVTL
   CHS1_protein  GLTFHLLKDVPGLISKNIEKALVEAFQPLGISDYNSIFWIAHPGGPAILDQVEAKLGLKPEKMEATRHVLSEYGNMSSACVLFILDQMRKKSIENGLGTTGEGLDWGVLFGFGPGLTVETVVLRSVTL
      Consensus  GLTFHLLKDVPGLISKNIEKALVEAFQPLGISDYNSIFWIAHPGGPAILDQVEAKLGLKPEKMEATRHVLSEYGNMSSACVLFILDQMRKKSIENGLGTTGEGLDWGVLFGFGPGLTVETVVLRSVTl
```

Supplemental Data Cho et al.

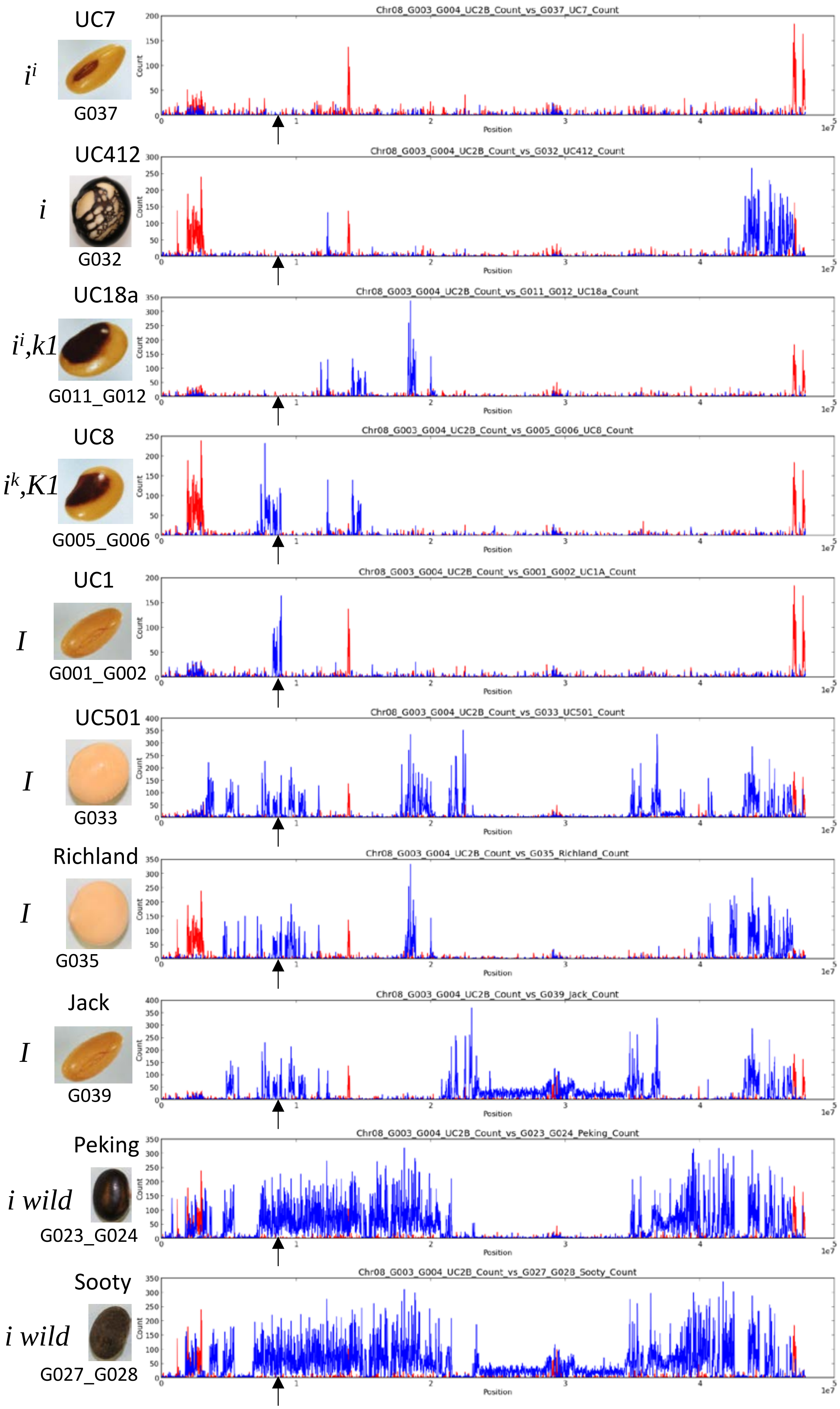

Supplemental Figure 5. Legend continued on next page.

Supplemental Figure 5. Two-Way Comparisons of SNPs and Small Insertions and Deletions in UC2 Clark (i^i Yellow Seed Coat, Black Hilum) Versus Other Sequenced Isolines or Cultivars with Different Alleles of the *I* Locus**.**

The seed phenotypes and genotypes are shown on the left side of ten isolines or cultivars that were subjected to whole genome resequencing and alignment to the Wm82.a2 reference genome. The number of variants on chr 8 for each isoline or variety were pooled into bins of 10,000 nt and plotted on the y-axis versus position of the bins on the x-axis. The number of variants per binned position of the ten sequenced isolines or cultivars (denoted by the blue lines) was compared to the number of variants in the standard Clark UC2 line (i^i yellow seed coat, black hilum) which is represented by the same red line in each graph. The black arrow underneath marks the SNP peaks found in the *I* (G001_G002_UC1) and i^k (G005_G006_UC8) isolines at 8.25 to 8.65 million which is the same region as shown in Fig. 5. It is clear in these two isoline comparisons that the SNP peaks in this region were introgressed during the repetitive backcrossing to form these two isolines. Other regions of considerable variation are found in the three cultivars that carry dominant *I* alleles (UC501, Richland, and Jack) as well as in the recessive *i* wild varieties Peking and Sooty.

Supplemental Data Cho et al.

Supplemental Table 1. Primer and Reporter Sequences Used for Digital PCR to Detect Copy Number of Highly Similar CHS Genes Which Comprise the *I* Locus Region on Chromosome 8

Target Gene	Primer	Sequence (Length)	CHS	1	2	3	4	5	6	7	8	9
CHS	Forward	TGCTTGTCGTTTGTTCTGAGATCA (24)	# of mismatch	0	1	0	0	0	4	3	2	0
	Reverse	GGCTATCAAGATGGGTGTCAGTTG (24)		0	0	0	0	0	4	6	6	0
	Reporter	CCGCAGTCACATTCC (15)		1	4	0	0	0	4	2	2	0

Reference gene	Primer	Sequence (Length)	Lectin	1	2
Lectin	Forward	TTTAGCTGAAGCAAAGCAATGG (22)	# of mismatch	0	Not aligned
	Reverse	GTTAGGGAGAGAGATACAACCACATTC (27)		0	Not aligned
	Reporter	CAAAGTTGAAAACCC (15)		0	Not aligned

The # of mismatches shows a count of mismatches between the sequence of the primers and the sequence of the target or reference genes. As shown, the *CHS* forward and reverse primers have no mismatches for the *CHS* genes on chr 8 (1, 3, 4, 5, and 9) but numerous mismatches for *CHS* genes *6*, *7,* and *8* and the reporter has numerous mismatches for *CHS* genes *2*, *6*, *7*, *8*. The seed lectin gene *Le1* (Vodkin et al., Cell 1983) was used as a reference. Copy number of the target genes = adjusted signal of target gene / adjusted signal of the single copy reference gene.
