# Supplementary material for: Nonallelic homologous recombination events responsible for copy number variation within an RNA silencing locus
Source: Plant Direct. 2019 Aug 27;3(8):e00162. doi: 10.1002/pld3.162 (PMC6710647; doi:10.1002/pld3.162)
Supplement: Supplementary file 6 [file PLD3-3-e00162-s006.pdf]

Dear Colleagues,

Thank you for the decision and the helpful reviews of our manuscript and reviews transferred from Plant Cell to Plant Direct. Our responses are in blue type and outline where changes have been made in the manuscript. In addition to the changes below, we have made a few formatting changes and added one sentence to the Figure 5 legend to clarify phenotypes and one additional reference about the seed phenotypes. All changes in the manuscript are in track changes and also highlighted.

Reviewer #1 (Comments for the Author):

Post transcriptional gene silencing of Chalcone synthase (CHS) by endogenous siRNAs produced by the I locus regulated seed coat color in soybean. Cho et al have compared copy number variation and single nucleotide polymorphisms among 15 soybean cultivars that carry different alleles of the I locus. Digital PCR was used to evaluate copy numbers of CHS genes specifically in the siRNA generating I locus. Amplicon sequencing of a portion of the I locus in lines with reduced copy numbers and whole genome re-sequencing of all the lines were used to determine the nature of deletion and changes in genetic sequence respectively. The work has identified gaps in the current genome assemblies of soybean and reveals a rare example of a potentially non-allelic homologous recombination event in plants. This recombination led to large deletions in the siRNA generating region (leading to light seed coat color). The data are presented clearly and figure legends are detailed and self-sufficient. The findings are likely to be interest to soybean breeders and some genome biologists. I have the following comments/concerns on the manuscript.

Comment 1. Digital PCR provides clear grouping of I, ii and mutant derivatives i (UC9, W55, W130) although W55 appears to have large variations in copy number. Is this a technical artifact? Can the authors generate supporting data using alternate approaches?

Response: In Figure 2, one of the three digital PCR biological repeats was a significant outlier for the W55 sample indicating a higher copy number than the other two lines in this grouping which were UC9 and W130. Based on further evidence shown in Figures 3 and 4, this one technical repeat outlier is very likely from a sampling or labeling error. The W55 sample is one of the mutant lines that were subjected to whole genome sequencing and the data clearly show a large deletion for this particular mutation in Figure 3a (Williams 55 is the same as W55) which includes a number of the CHS genes. Finally, Figure 4c shows the sequence of the 7.9 kb amplicon from W55, demonstrating that the recombination event between the distal two CHS genes eliminated the intervening CHS copies. Thus, we are confident that the two repeats that show a low copy number, and not the outlier for the digital PCR data, represent the W55 line.

Comment 2. It is clear that ii > i mutations resulted in loss of siRNA-generating CHS copies in UC9, W55, and W130, and a smaller deletion in UC142. No clear explanation is provided for the relationship between copy numbers and phenotypes of other groups. The authors did whole genome resequencing of the lines, but observed complete base coverage w/o evidence for deletions in these other lines with a dark seed coat phenotype or the apparent copy number

variation between I and ii. The statement in lines 331-333 seems to indicate that reads from the repeat regions may not be distinguishable. Is it possible to amplify smaller fragments of the repeat regions to obtain clues (size difference) or perform amplicon sequencing? Did the authors closely evaluate alignments for potential variations between the duplicate copies of each CHS gene in I locus?

Response: This system illustrates the difficulty of working with highly repeated sequence regions in that primer choices for determining the exact structure are severely limited. As illustrated in our browser track lines in Figures 1, 3, and 6, the blue lines represent identical and red lines nearly identical 10 to 20 kb regions. As we indicated in the original manuscript on page 9, in lines 282-293, we tried unsuccessfully with numerous primer sets to get amplicon fragments that would span the breakpoint of the smaller deletion in UC412. However, since there are regions of 100% similarity within the CHS inverted repeat region for spans of over 10,000 bases, there are few good choices for amplifying fragments less than that size. Ironically, our success in amplifying fragments that span the breakpoint region of the larger deletions found in the W55, UC9, and W130 mutations resulted from the naturally occurring NAHR removal of most of the *CHS* genes and also of the second primer binding region to the epimerase gene (shown by the red arrows in Figure 3a) resulting in successful amplification of a PCR fragment.

Repetitive regions of larger sizes also cause difficulty in extracting breakpoints for paired-end whole genome sequences when the pairs are an average size of approximately 500 bases which is standard. As stated in the Introduction and in the display of the two JGI reference genome assemblies shown in Figure 1a and 1b, these automated assemblies did not represent the i^i allele accurately but instead had inversions and/or gaps. The BAC sequences that we had reported earlier by traditional Sanger sequencing of overlapping subclones and primer walking were more accurate for the *I* locus as shown in Figure 1c. As we discussed in the manuscript, longer read sequencing technologies may be applicable to the *I* locus region in the future.

On the other hand, the digital PCR results in Figure 2, have value in assessing the number of copies for very similar sequences as with the CHS genes and led to classification of the mutations in various groups by copy number. Thus, taken together, the two technologies reinforce each other.

Comment 3. Legend in Figure 5 indicates that SNPs were identified based in W82.a2 reference genome (non-modified). The authors state that there are gaps in this assembly and they modified it based on BAC sequences. It is not clear to me why the authors did not use the one they modified version for SNP identification.

Response: Since the modified gene region is only different around the inverted repeat region of the *I* locus, we wanted to stay with the currently available Williams 82.a2 genome for our whole genome analyses of SNP variants so that the position numbers would be the same on chromosome 8. Either way, the conclusion would be the same that the SNP density for the I locus is very different between the i^i, *I*, and wild *i* alleles. This finding has implications for breeding using different germplasm lines.

Comment 4. While the focus on this work is the I locus alleles, evaluation of the stilbene branch

genes for potential duplication and presence in repeat gene regions might reveal evolutionary relationships regarding siRNA-mediated regulation of these gene families.

Response: That would be an interesting project, and we can do that with current small RNA and RNA-Seq data, but it would be a separate project if there were to be any positive indication for such an RNAi regulation.

5. Please add kb scale bars in Figures 1, 3a, 4a and 6a.

Response: The kb positions on the gene tracks in Figure 1e, 3a, 4a, and 6a are found below the alignment track at the vertical bar positions. We have indicated this more clearly in the legends and that the vertical bars denote 20 kb segments.

Reviewer #2 (Comments for the Author):

This study demonstrates the role of NAHR in generating mutant alleles in the locus controlling seed coat color in soybean. As the authors indicate, this is likely the first direct observation, albeit by pedigree, of NAHR in plants, though it has described at length in yeast and human. This continues the corresponding author's work on the genetics of seed coat color in soybean which includes a complex arrangement of duplicated genes that lead to interfering RNAs.

The data and analysis are thorough and I don't see any major, or minor, flaws, but I am somewhat concerned about how much general interest it will generate. The only suggestion would be to have a model for the NAHR to make it easier for the reader to understand how the alleles are generated.

Minor comment: p15 and 16, Lye and Peruganan 2019 should be Lye and Purugganan.

Response to Reviewer #2: Because of the length of the paper already, and the fact that many gene tracks with the repetitive gene region have already been displayed in each figure, we do not feel that a general model of non-allelic homologous recombination would add significantly. Figure 4 is the critical figure showing the data supporting the hybrid gene.
We have corrected the spelling indicated.

Reviewer #3 (Comments for the Author):

The authors examined copy number variation (CNV) of the *CHS* gene within a segment of chromosome 8 in the soybean genome that contains the *I* locus responsible for phenotypic changes in seed coat color. CNV analysis and whole-genome re-sequencing of 15 cultivars containing different alleles of the I locus identified changes in *CHS* gene copy number associated with the alleles. Furthermore, the authors observed NAHR events between *CHS* genes located within the segmentally duplicated region on chromosome 8 that resulted in the observed allelic variation at the *I* locus resulting in a hybrid *CHS* gene.

The work is well-written and characterization/analysis of the *l* locus was thorough. The reviewer appreciates the use of the seed coat phenotype associated with each allele and cultivar in the

figures. The finding was significant in furthering our understanding of the regulation of phenotypic changes (i.e., seed coat color) at the DNA level associated with a domestication trait. While the science is solid, I am concerned with the general interest of the work.

Minor comments:

1. On line 544, the authors stated "three to four biological replicates" were used but the legend in Figure 2B associated with the analysis indicated "three to six replicates".

Response: We changed page 15 to read “three to six” which is correct.

2. Figure 6 legend part (B-C). The text should read "four flanking genes are graphed in part (B)."

Response: This has been corrected.

3. On line 614, the authors stated "Supplemental Data Sets 1-5" but there are only Data Sets 1-4.

Response: This has been corrected to read Supplemental Data Sets 1-4.

Reviewer #4 (Comments for the Author):

Non-allelic homologous recombination events responsible for copy number variation within an RNA silencing locus (TPC2019-RA-00457) by Cho et al.

The authors reviewed current and past genome assemblies and identified gaps and sequence re-arrangements resulting from difficulties assembling highly repetitive loci. They aligned this region to a previously published BAC sequence and modified the locus sequence accordingly. Sequence data mapped correctly to the modified sequence. The authors next used digital PCR to quantify CHS copy number in soybean lines with different alleles of the I locus and found copy numbers in three black seed lines (UC9, W55 & W130). A 8kbp amplicon within the I locus was generated in these lines and it was sequenced to confirm a large ~138 kbp deletion resulting in a novel hybrid CHS gene comprised of both CHS5 and CHS1 genes called CHS5:1. They used amplicon sequence data to show the formation of this gene was generated by non-allelic homologous recombination. Using whole genome sequencing of yellow and black seeded lines they show the SNP variations of the I locus in different alleles.

Importance of findings:
Demonstrated the black seed phenotype from the i mutation resulted from a deletion repaired by non-allelic homologous recombination.
The authors corrected the mis-annotated genome assembly at the I locus.

Quality of experiments:
Excellent

Points in favor:

Well written and an enjoyable read

Points detracting:
The authors claim in the summary that expansion of CHS gene formed an inverted CHS repeat that spawned production of CHS siRNAs resulting in yellow seed coats. This claim is not a novel finding as it has been presented in previous work by Tuteja et al. 2009, Cho et al. 2017 etc.

Response: We have rewritten our summary paragraph to clarify what was background of the system and what we reported in this manuscript.
